# Tethering of SCF^Dia2^ to the Replisome Promotes Efficient Ubiquitylation and Disassembly of the CMG Helicase

**DOI:** 10.1016/j.cub.2015.07.012

**Published:** 2015-08-31

**Authors:** Timurs Maculins, Pedro Junior Nkosi, Hiroko Nishikawa, Karim Labib

**Affiliations:** 1 Cancer Research UK Manchester Institute, University of Manchester, Wilmslow Road, Manchester M20 4BX, UK; 2 MRC Protein Phosphorylation and Ubiquitylation Unit, Sir James Black Centre, College of Life Sciences, University of Dundee, Dow Street, Dundee DD1 5EH, UK

## Abstract

Disassembly of the Cdc45-MCM-GINS (CMG) DNA helicase, which unwinds the parental DNA duplex at eukaryotic replication forks, is the key regulated step during replication termination but is poorly understood [1, 2]. In budding yeast, the F-box protein Dia2 drives ubiquitylation of the CMG helicase at the end of replication, leading to a disassembly pathway that requires the Cdc48 segregase [3]. The substrate-binding domain of Dia2 comprises leucine-rich repeats, but Dia2 also has a TPR domain at its amino terminus that interacts with the Ctf4 and Mrc1 subunits of the replisome progression complex [4, 5], which assembles around the CMG helicase at replication forks [6]. Previous studies suggested two disparate roles for the TPR domain of Dia2, either mediating replisome-specific degradation of Mrc1 and Ctf4 [4] or else tethering SCF^Dia2^ (SCF [Skp1/cullin/F-box protein]) to the replisome to increase its local concentration at replication forks [5]. Here, we show that SCF^Dia2^ does not mediate replisome-specific degradation of Mrc1 and Ctf4, either during normal S phase or in response to replication stress. Instead, the tethering of SCF^Dia2^ to the replisome progression complex increases the efficiency of ubiquitylation of the Mcm7 subunit of CMG, both in vitro and in vivo. Correspondingly, loss of tethering reduces the efficiency of CMG disassembly in vivo and is synthetic lethal in combination with a disassembly-defective allele of *CDC48*. Residual ubiquitylation of Mcm7 in *dia2-ΔTPR* cells is still CMG specific, highlighting the complex regulation of the final stages of chromosome replication, about which much still remains to be learned.

## Highlights

• Replisome tethering of SCF^Dia2^ promotes efficient ubiquitylation of the CMG helicase
• Loss of tethering and mutation of Cdc48 cause synthetic CMG disassembly defects

## Graphical Abstract

**Figure:**
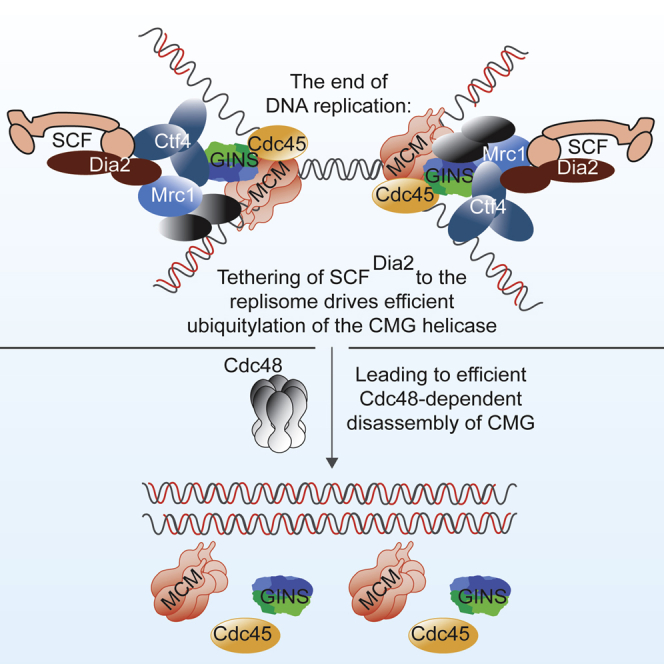

## Teaser

Disassembly of the CMG helicase is the key regulated step at the end of chromosome replication in eukaryotes, and in budding yeast, this is driven by the ubiquitin ligase SCF^Dia2^ and the Cdc48 segregase. SCF^Dia2^ is tethered to the replisome, and Maculins et al. show that this connection controls the efficiency of CMG ubiquitylation and disassembly.

## Results and Discussion

### CMG Disassembly Explains the Apparent Instability of Replisome-Associated Mrc1 and Ctf4

**Figure 1:**
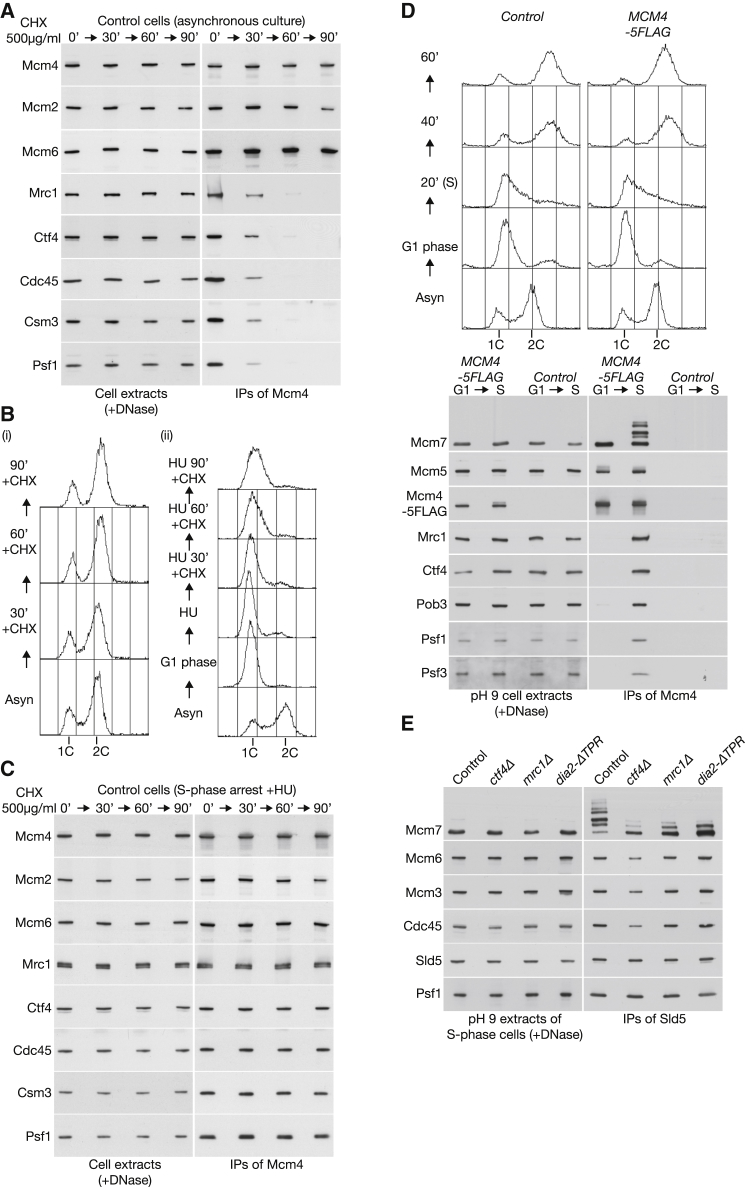
Tethering of SCF^Dia2^ to the Replisome Progression Complex Increases the Efficiency of CMG Ubiquitylation In Vitro (A) An asynchronous culture of *MCM4-5FLAG MRC1-18MYC* cells (YGDP219) was grown at 30°C, before addition of 500 μg/ml cycloheximide for the indicated times. Cell extracts were treated with DNase before immunoprecipitation of Mcm4-5FLAG and detection of the indicated proteins by immunoblotting. (B) (i) Flow cytometry analysis from the same experiment. (ii) The same strain as above was arrested in G1 phase and then released into S phase for 60 min in the presence of 0.2 M hydroxyurea. Cycloheximide was added for the indicated times and samples processed as before. (C) The samples from (Bii) were processed as in (A). (D) Control cells (YTM325) and *MCM4-5FLAG* (YTM326) were synchronized at 30°C in the G1 phase of the cell cycle by addition of mating pheromone, before release into S phase for 20 min. DNA content was monitored by flow cytometry (upper panels). “pH 9 cell extracts” were then prepared as described in the Supplemental Experimental Procedures and incubated with magnetic beads coupled to anti-FLAG monoclonal antibody. The immunoprecipitated proteins were then monitored by immunoblotting (lower panels). (E) Control (YASD375), *ctf4Δ* (YTM403), *mrc1Δ* (YLG31), and *dia2-ΔTPR* (YTM265) were synchronized in early S phase as above, before immunoprecipitation of TAP-Sld5 from pH 9 cell extracts on IgG beads. See also Figures S1 and S2.

Previous work showed that the association of budding yeast Mrc1 and Ctf4 with the Cdc45-MCM-GINS (CMG) helicase was lost in control cells, but not in *dia2Δ*, when cycloheximide was used to inhibit protein synthesis in asynchronous cell cultures [4]. This was taken as evidence that SCF^Dia2^ specifically ubiquitylates the fraction of Mrc1 and Ctf4 that is incorporated into the replisome progression complex at replication forks. We repeated the same experiment with control cells expressing *DIA2* by immunoprecipitating the Mcm4 helicase subunit from cell extracts after addition of cycloheximide. Whereas Mcm4 still associated with the remaining subunits of the Mcm2-7 complex in cycloheximide-treated cells, association with all other RPC subunits was lost (Figure 1A). Rather than reflecting the specific degradation of RPC-associated Mrc1 and Ctf4, these data thus indicated that the RPC is no longer present when control cells are treated with cycloheximide. A simple explanation for this is provided by the fact that protein synthesis is required for G1 phase cells to enter S phase, but S phase cells can complete DNA replication without ongoing protein synthesis [7–9]. Consistent with this view, flow cytometry data from the same experiment indicated that the S phase population of cells was lost upon addition of cycloheximide to the asynchronous cell culture (Figure 1Bi). Cycloheximide should thus block the assembly, but not the disassembly, of the RPC.

To confirm that loss of the RPC in cycloheximide-treated cells reflects CMG disassembly during the completion of chromosome replication, we arrested cells with hydroxyurea in early S phase, prior to cycloheximide treatment. In contrast to the above experiment, the association of Mcm4 with all other RPC components including Ctf4 and Mrc1 was preserved in hydroxyurea-arrested cells upon treatment with cycloheximide (Figure 1C), reflecting the stable persistence of the replisome at stalled replication forks. Moreover, the same was true when cells lacking the Mec1 checkpoint kinase were arrested with hydroxyurea and then treated with cycloheximide, indicating that the persistent association of Mrc1 and Ctf4 with the RPC in hydroxyurea-arrested control cells did not reflect the inhibition of SCF^Dia2^ by the S phase checkpoint pathway (Figure S1A).

Subsequently, we treated an asynchronous culture of *dia2Δ* cells with cycloheximide and observed the persistent association of Mcm4 with all tested RPC components including Mrc1 and Ctf4 (Figure S1B). These data are explicable by the failure of *dia2Δ* cells to disassemble the CMG helicase at the end of S phase [3].

Finally, we directly examined RPC ubiquitylation in an extract of S phase yeast cells, using conditions that we had previously shown to support efficient in vitro ubiquitylation of CMG on its Mcm7 subunit, dependent upon SCF^Dia2^ and the Cdc34 ubiquitin-conjugating enzyme [3]. Whereas the in vitro ubiquitylation of CMG was easily detected in these “pH 9 cell extracts,” we did not detect ubiquitylation of the associated RPC subunits including Mrc1 and Ctf4 (Figure 1D). Taken together, the preceding experiments reflect the disassembly of the CMG helicase during replication termination in control cells and the failure of CMG disassembly in *dia2Δ* cells but do not provide evidence for the replisome-specific ubiquitylation of Mrc1 and Ctf4 by SCF^Dia2^.

### Tethering of SCF^Dia2^ to the Replisome Progression Complex Increases the Efficiency of CMG Ubiquitylation In Vitro

To examine whether ubiquitylation of the CMG helicase is dependent upon tethering of SCF^Dia2^ to the replisome progression complex (Figure S2), we compared the ability of S phase extracts of control cells *ctf4Δ*, *mrc1Δ*, or *dia2-ΔTPR* to support in vitro CMG ubiquitylation. As seen in our previous study [3], ubiquitylation of Mcm7 was restricted to the specific fraction that is present in the CMG helicase, which we isolated by immunoprecipitation of the Sld5 subunit of GINS (Figure 1E). In control cell extracts, almost all CMG complexes had ubiquitylated Mcm7 under these conditions, producing a ladder of modified Mcm7 bands in which unmodified Mcm7 was only a minor form (Figure 1E; IPs of Sld5; control). Although ubiquitylated Mcm7 could still be detected when CMG was isolated from extracts of *ctf4Δ*, *mrc1Δ*, or *dia2-ΔTPR*, ubiquitylation was much reduced in all three cases compared to the control (Figure 1E). These findings indicated that the tethering of SCF^Dia2^ to the RPC, by interaction of the TPR of Dia2 with Mrc1 and Ctf4, is important for the efficiency of CMG ubiquitylation of CMG in vitro.

**Figure 2:**
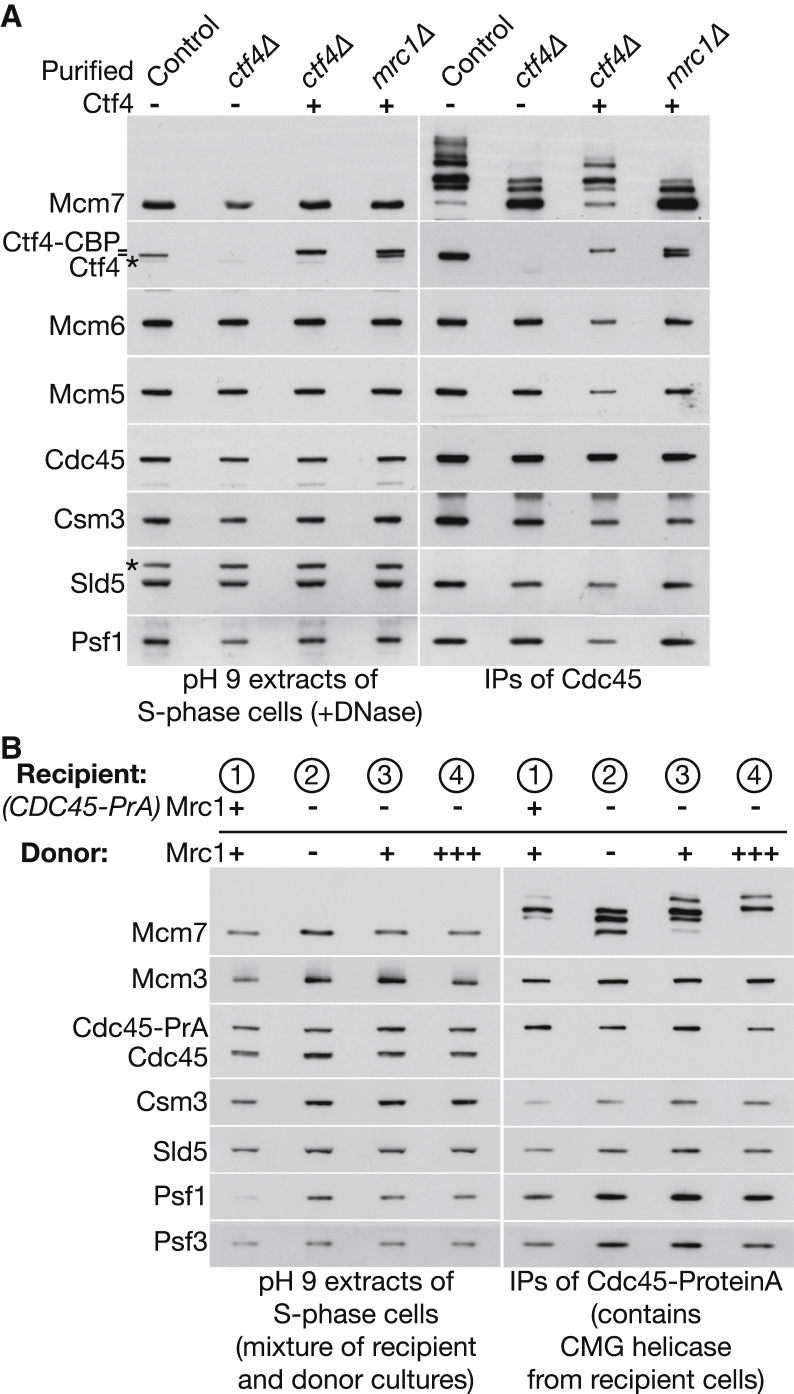
The CMG Ubiquitylation Defects of *ctf4Δ* and *mrc1Δ* Can Be Rescued In Vitro (A) S phase cell extracts of control (YTM401), *ctf4Δ* (YTM438), and *mrc1Δ* (YTM440) were prepared at pH 9 as above and complemented with buffer or purified Ctf4 as indicated, before immunoprecipitation of Cdc45-ProteinA. The indicated proteins were then monitored by immunoblotting. Asterisks denote non-specific bands. (B) To test for in vitro rescue of the ubiquitylation defect of *mrc1Δ* cell extracts, we synchronized the indicated *CDC45-ProteinA* “recipient strains” (1, YTM401; 2–4, YTM440) and *CDC45* “donor strains” (1–4, YSS3, YPNK314, YSS3, and YPNK342, respectively) in S phase at 30°C. Each of the indicated pairs of recipient and donor cultures were then mixed and used to prepare a single cell extract at pH 9 as above. After digestion of chromosomal DNA, the CMG helicase from recipient cells was isolated by immunoprecipitation of its ProteinA-tagged-Cdc45 subunit.

To confirm that loss of tethering reduced the capacity of SCF^Dia2^ to drive in vitro ubiquitylation of the CMG helicase, we repeated the above experiment with S phase extracts of control, *ctf4Δ*, or *mrc1Δ* and then complemented the extracts with buffer or with purified Ctf4 protein. Critically, addition of purified Ctf4 to the *ctf4Δ* extract restored the efficiency of ubiquitylation, producing a very similar pattern to the control extract (with di-ubiquitylated Mcm7 being the predominant form in the isolated CMG material), whereas addition of Ctf4 to an extract of *mrc1Δ* cells had no effect (Figure 2A). Similarly, we showed that the CMG ubiquitylation defect of *mrc1Δ* extracts could be rescued in vitro by mixing with extracts of cells that expressed Mrc1. We synchronized “recipient” cultures of *CDC45-ProteinA* in S phase alongside “donor cultures” expressing untagged *CDC45* and then mixed cultures as indicated in Figure 2B, before making cell extracts and isolating “‘recipient CMG” by immunoprecipitation of Cdc45-ProteinA. A donor extract expressing Mrc1 was able to rescue the in vitro ubiquitylation defect of an *mrc1Δ* recipient extract (Figure 2B, sample 3), whereas an extract overexpressing Mrc1 further enhanced the ubiquitylation of CMG (Figure 2B, sample 4). These findings demonstrate that the TPR-dependent tethering of SCF^Dia2^ to the RPC serves to increase the efficiency of CMG ubiquitylation in vitro.

### Tethering of SCF^Dia2^ to the Replisome Progression Complex Is Important for Efficient CMG Ubiquitylation In Vivo

**Figure 3:**
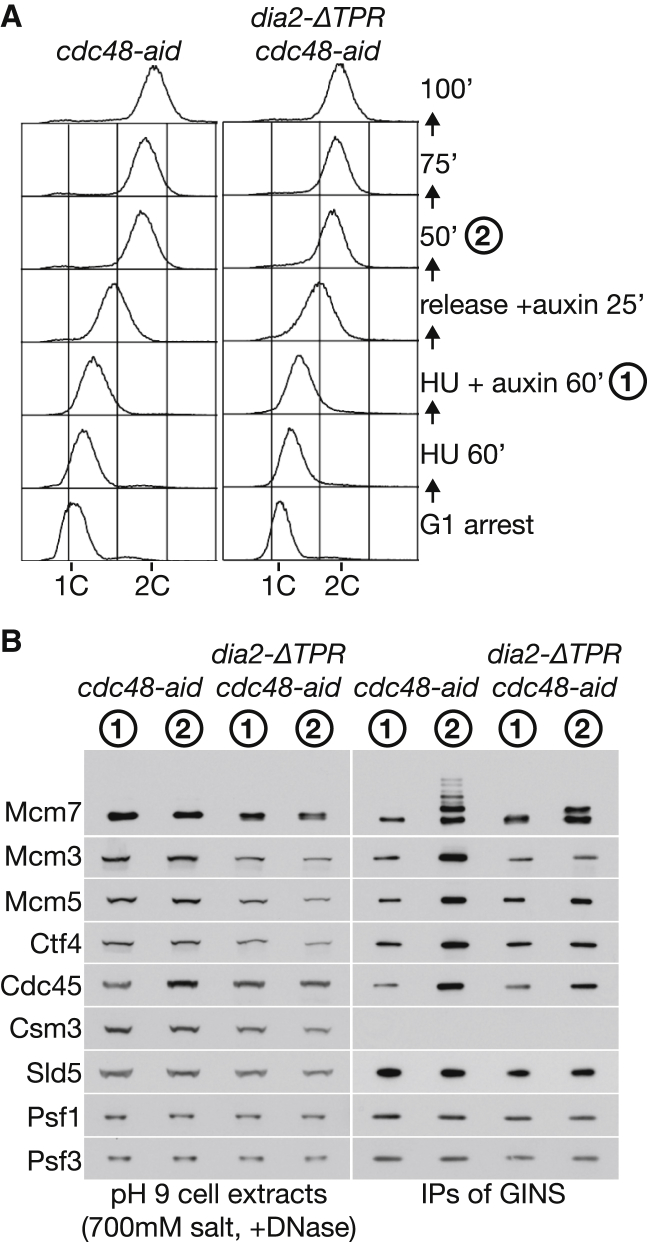
Tethering of SCF^Dia2^ to the Replisome Progression Complex Increases the Efficiency of In Vivo CMG Ubiquitylation at the End of S Phase (A) *cdc48-aid* (YMM228) and *cdc48-aid dia2-ΔTPR* (YPNK334) were synchronized in G1 phase at 30°C and then released into S phase for 60 min in the presence of 0.2 M hydroxyurea. For depletion of Cdc48-aid, 0.5 mM auxin was added for 60 min, before release into fresh medium containing auxin but lacking hydroxyurea. DNA content was monitored by flow cytometry, and samples were taken at the indicated times (“1” and “2”) to prepare pH 9 cell extracts containing 700 mM salt. (B) CMG helicase was isolated as above by immunoprecipitation of TAP-tagged Sld5 subunit.

Ubiquitylation of the CMG helicase is restricted to the end of chromosome replication in vivo, when it is coupled rapidly to Cdc48-dependent disassembly [3]. For visualization of ubiquitylated CMG in vivo, it is necessary to inactivate Cdc48 before cells terminate DNA replication and then prepare “high salt” extracts that block the in vitro ubiquitylation of the CMG helicase. In order to assess the contribution of replisome tethering of SCF^Dia2^ to CMG ubiquitylation in vivo, we synchronized *cdc48-aid* and *dia2-ΔTPR cdc48-aid* cells (aid [auxin inducible degron]) in early S phase and then depleted Cdc48-aid, before allowing cells to proceed with chromosome replication (Figure 3A). As shown in Figure 3B, in vivo ubiquitylation of the Mcm7 subunit of CMG could still be detected at the end of S phase in *dia2-ΔTPR cdc48-aid* cells but was markedly reduced. These data indicate that the tethering of SCF^Dia2^ to the RPC increases the efficiency of CMG ubiquitylation at the end of chromosome replication in budding yeast.

### Impaired Ubiquitylation of CMG in *dia2-ΔTPR* Cells Produces a Defect in CMG Disassembly

Cells lacking Dia2 have a very high rate of genome instability, are unable to grow at low temperatures, and are sensitive to DNA-damaging agents that perturb the progression of DNA replication forks [5, 10–12]. Dia2 drives the disassembly of the CMG helicase at the end of chromosome replication so that the absence of Dia2 causes CMG to persist into G1 phase of the next cell cycle [3], but at present, it is not known how this defect is linked to the other phenotypes of *dia2Δ* cells.

**Figure 4:**
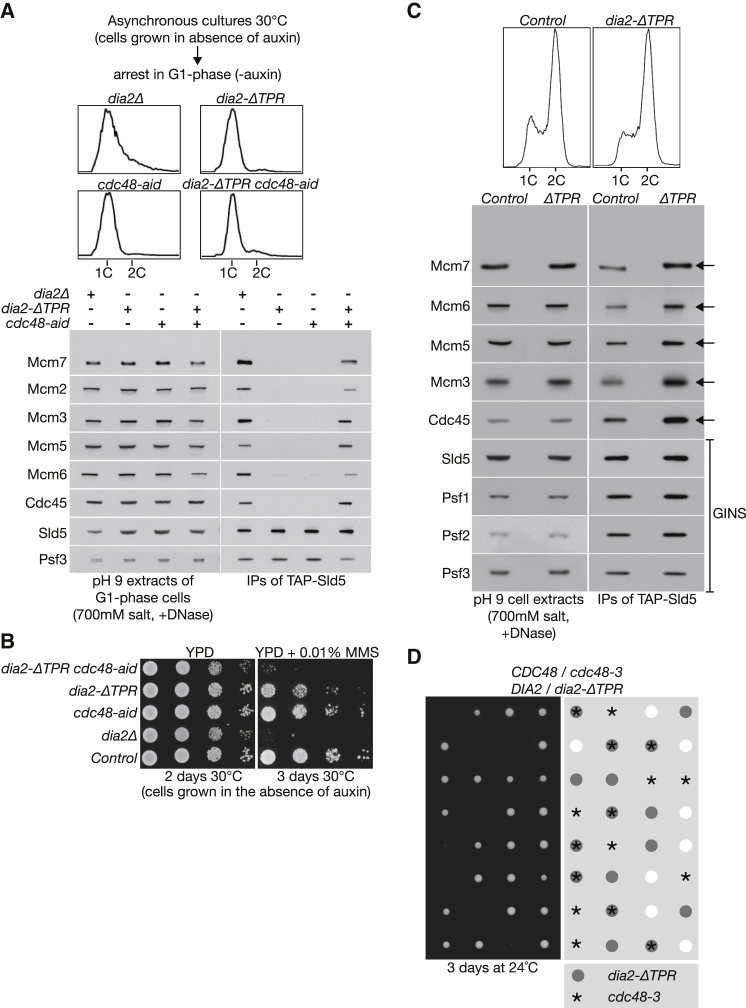
The CMG Disassembly Defect of *dia2-ΔTPR* Is Augmented by Mutations in *CDC48* (A) *dia2Δ* (YHM130), *dia2-ΔTPR* (YTM265), *cdc48-aid* (YMM228), and *dia2-ΔTPR cdc48-aid* (YPNK334) were arrested in G1 phase at 30°C, in medium lacking auxin (permissive conditions for *cdc48-aid*), before isolation of TAP-Sld5 from “high-salt pH 9 cell extracts.” (B) Serial dilutions of the indicated strains were grown on rich medium (YPD) in the absence or presence of the DNA-damaging agent methyl methanesulfonate. (C) Asynchronous cultures of control (YASD375) and *dia2-ΔTPR* (YTM265) were grown at 30°C, before isolation of TAP-Sld5 from high-salt pH 9 cell extracts as above. The arrows denote the increased association of Cdc45 and Mcm2-7 proteins with GINS in extracts of asynchronous *dia2-ΔTPR* cells. (D) The diploid strain *CDC48/cdc48-3 DIA2/dia2-ΔTPR* was sporulated and subjected to tetrad analysis. The germinated spores were grown for 3 days at 24°C before imaging. See also Figures S3 and S4.

Although CMG was not detected during G1 phase in *dia2-ΔTPR* cells grown at 30°C, or in *cdc48-aid* cells grown at 30°C in medium lacking auxin (permissive conditions, in which the phenotype just reflects the C-terminal tag on Cdc48), we found that the combination of *dia2-ΔTPR* with *cdc48-aid* produced a synthetic defect in CMG disassembly that resembled the phenotype of *dia2Δ* cells at 30°C (Figure 4A). Moreover, the *dia2-ΔTPR cdc48-aid* strain also shared the sensitivity of *dia2Δ* cells to the DNA-damaging agent methyl methanesulfonate (Figure 4B; note that cells were grown in the absence of auxin). These findings suggested that *dia2-ΔTPR* cells have a partial defect in CMG disassembly, even though helicase disassembly is still completed by the end of the cell cycle. Accordingly, we found that asynchronous cultures of *dia2-ΔTPR* contained slightly more CMG helicase than control cells (Figure 4C; we cannot exclude that the activation of more origins during S phase might also contribute to this effect in *dia2-ΔTPR* cells).

We previously showed that the *cdc48-3* allele has a partial defect in CMG disassembly at the permissive temperature of 24°C [3]. Strikingly, we found that *dia2-ΔTPR* was synthetic lethal with *cdc48-3* at 24°C (Figure 4D), reminiscent of the cold-sensitive phenotype of *dia2Δ* cells [5]. Moreover, *ctf4Δ* was also synthetic lethal with *cdc48-3* at 24°C, whereas *mrc1Δ cdc48-3* showed a synthetic growth defect (Figure S3). In contrast, deletion of factors with other roles at defective replication forks, such as Top3 or Rad51, did not cause synthetic lethality with *cdc48-3* (Figure S3). Taken together, these findings indicate that tethering of SCF^Dia2^ to the RPC contributes to efficient disassembly of the CMG helicase at the end of chromosome replication in budding yeast.

CMG disassembly represents the key regulated step during replication termination, which drives replisome disassembly and must not occur prematurely [1, 2]. Although CMG ubiquitylation and Cdc48-dependent disassembly have been conserved from budding yeast to vertebrates, the mechanism and regulation of CMG disassembly are still very poorly characterized in all eukaryotes. Budding yeast SCF^Dia2^ is currently the only ubiquitin ligase that has been shown to drive CMG disassembly in any species and thus provides an important model system with which to study the underlying principles.

It seems likely that ubiquitylation is rate limiting for CMG disassembly, although this remains to be demonstrated by mapping and mutation of the ubiquitylation sites in Mcm7. It is clear that Mcm7 ubiquitylation is regulated in an exquisite fashion on many levels, both spatially and temporally. One key aspect is that ubiquitylation of Mcm7 only occurs in the context of the CMG helicase and thus is restricted to replication forks. Our findings in this study indicate that SCF^Dia2^ is preferentially targeted to the replisome progression complex, rather than simply to the CMG helicase itself. Tethering of SCF^Dia2^ to the RPC increases the efficiency of CMG ubiquitylation and involves the interaction of the TPR domain of Dia2 with both Ctf4 and Mrc1, which only come together in the context of the RPC. Nevertheless, the residual ubiquitylation of Mcm7 in cells that cannot tether SCF^Dia2^ to the RPC is also CMG specific (Figures 2 and 3). One possibility is that the leucine-rich repeats of Dia2 target the ligase to Mcm7 in a CMG-dependent manner that requires a structural change in the helicase during termination.

Factors that drive the assembly of the CMG helicase during the initiation of replication, such as the Cdc7 kinase or the TopBP1 adaptor protein, are currently being pursued as targets for new anti-cancer therapies in tumors that retain inherent defects in chromosome replication [13–15]. It will be interesting to explore the potential of CMG disassembly for future therapies, and it will thus be important to determine the ubiquitin ligase(s) driving CMG disassembly in human cells and other eukaryotic species. Orthologs of Dia2 are present in other yeasts [16], including fission yeast Pof3 that appears to use its TPR domain to target Ctf4 in a manner analogous to budding yeast Dia2 (Figure S4). Moreover, a small-molecule inhibitor of cullin neddylation blocks CMG ubiquitylation at the end of DNA replication in frog egg extracts [17]. Nevertheless, homologs of Dia2 have yet to be identified in higher eukaryotes, and it is possible that an unrelated E3 ligase ubiquitylates CMG at the end of chromosome replication in other species. Functional screens for factors driving CMG disassembly in higher eukaryotes will be an important challenge for future studies.

## Author Contributions

T.M. performed the experiments in Figures 1, 2A, S1, and S3. P.J.N. carried out the experiments in Figures 2B, 3, 4, and S2. H.N. performed the experiments in Figure S4. K.L. designed the project in collaboration with T.M., P.J.N., and H.N. and wrote the manuscript.

## Supplemental Information

Document S1. Supplemental Experimental Procedures, Figures S1–S4, and Table S1

Document S2. Article plus Supplemental Information
