# Supplementary material for: Tethering of SCFDia2 to the Replisome Promotes Efficient Ubiquitylation and Disassembly of the CMG Helicase
Source: Curr Biol. 2015 Aug 31;25(17):2254–9. doi: 10.1016/j.cub.2015.07.012 (PMC4562905; doi:10.1016/j.cub.2015.07.012)
Supplement: Document S1. Supplemental Experimental Procedures, Figures S1–S4, and Table S1 [file mmc1.pdf]

Current Biology
Supplemental Information

Tethering of SCF^{Dia2} to the Replisome Promotes Efficient Ubiquitylation and Disassembly of the CMG Helicase

Timurs Maculins, Pedro Junior Nkosi, Hiroko Nishikawa, and Karim Labib

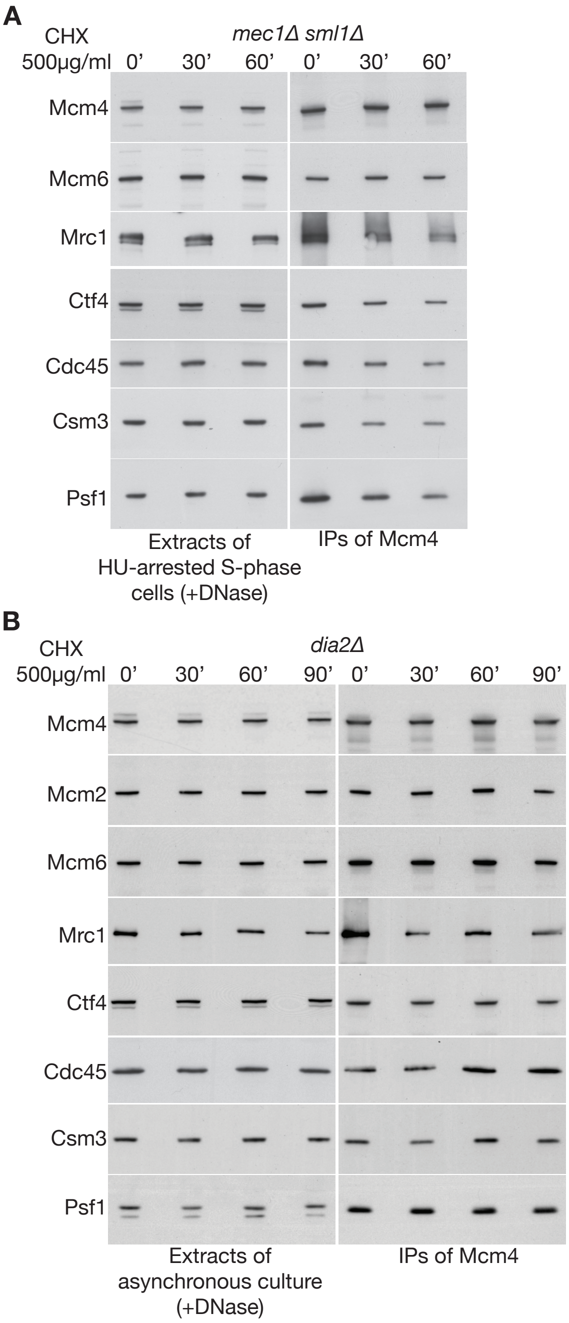
A
CHX
500µg/ml
mec1Δ sml1Δ
0’ 30’ 60’ 0’ 30’ 60’
Mcm4
Mcm6
Mrc1
Ctf4
Cdc45
Csm3
Psf1
Extracts of HU-arrested S-phase cells (+DNase)
IPs of Mcm4
B
CHX
500µg/ml
dia2Δ
0’ 30’ 60’ 90’ 0’ 30’ 60’ 90’
Mcm4
Mcm2
Mcm6
Mrc1
Ctf4
Cdc45
Csm3
Psf1
Extracts of asynchronous culture (+DNase)
IPs of Mcm4

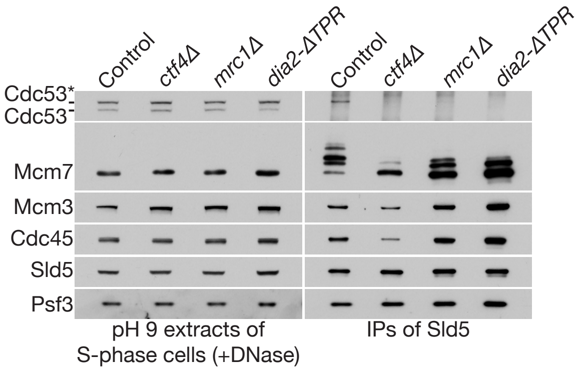
Control
ctf4Δ
mrc1Δ
dia2-ΔTPR
Control
ctf4Δ
mrc1Δ
dia2-ΔTPR
Cdc53*
Cdc53
Mcm7
Mcm3
Cdc45
Sld5
Psf3
pH 9 extracts of
S-phase cells (+DNase)
IPs of Sld5

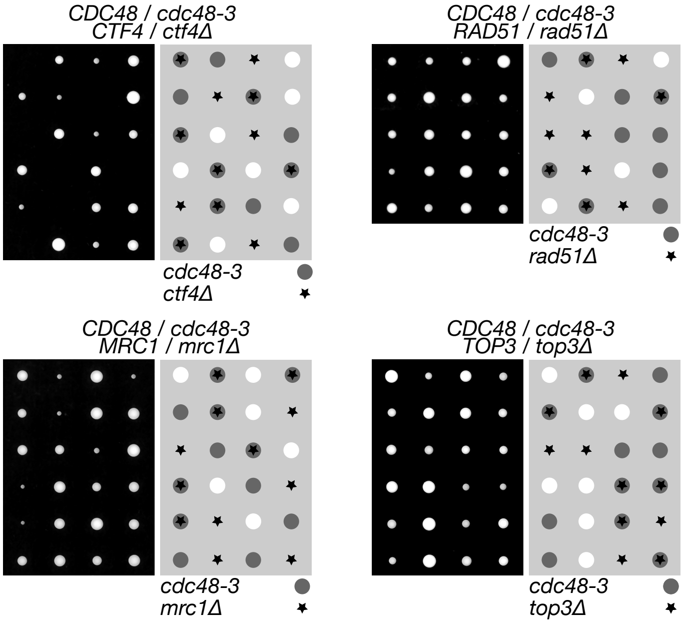
CDC48 / cdc48-3
CTF4 / ctf4Δ
cdc48-3
ctf4Δ
CDC48 / cdc48-3
RAD51 / rad51Δ
cdc48-3
rad51Δ
CDC48 / cdc48-3
MRC1 / mrc1Δ
cdc48-3
mrc1Δ
CDC48 / cdc48-3
TOP3 / top3Δ
cdc48-3
top3Δ

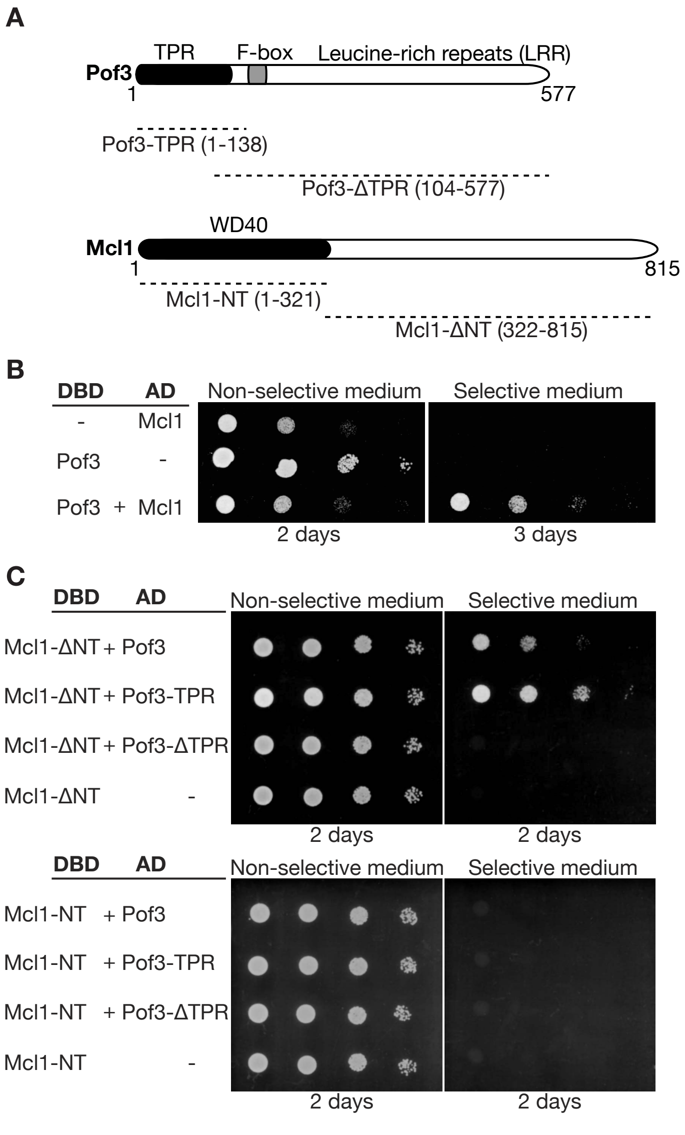

A
TPR
F-box
Leucine-rich repeats (LRR)
Pof3
1
577
Pof3-TPR (1-138)
Pof3-ΔTPR (104-577)
WD40
Mcl1
1
815
Mcl1-NT (1-321)
Mcl1-ΔNT (322-815)
B
DBD
AD
Non-selective medium
Selective medium
- Mcl1
Pof3 -
Pof3 + Mcl1
2 days
3 days
C
DBD
AD
Non-selective medium
Selective medium
Mcl1-ΔNT + Pof3
Mcl1-ΔNT + Pof3-TPR
Mcl1-ΔNT + Pof3-ΔTPR
Mcl1-ΔNT -
2 days
2 days
DBD
AD
Non-selective medium
Selective medium
Mcl1-NT + Pof3
Mcl1-NT + Pof3-TPR
Mcl1-NT + Pof3-ΔTPR
Mcl1-NT -
2 days
2 days

Figure S1 related to Figure 1

Mrc1 and Ctf4 still associate with the replisome when CMG disassembly cannot occur. (**A**) A *mec1Δ sml1Δ* strain (YGDP417) was arrested in G1-phase at 30°C and then released into S-phase in the presence of 0.2M hydroxyurea for 60', before addition of cycloheximide for the indicated times. Samples were then processed as above. (**B**) An asynchronous culture of *dia2Δ* cells (YTM179) was processed as in Figure S1A above.

Figure S2 related to Figure 1

Tethering of SCF^{Dia2} to the replisome is broken by mutation of Ctf4, Mrc1, or the TPR domain of Dia2. The experiment in Figure 1E was repeated, and the association of SCF^{Dia2} with the CMG helicase monitored by immunoblotting of the Cdc53 cullin subunit. Note that Cdc53 is present in two forms in cell extracts: a faster migrating form that represents unmodified Cdc53, and a slower migrating form corresponding to neddylated Cdc53 (marked Cdc53*). The latter associates preferentially with the CMG helicase. The association of SCF^{Dia2} with the replisome is dependent upon Ctf4, Mrc1 and the TPR domain of Dia2.

Figure S3 related to Figure 4

Synthetic interactions of *ctf4Δ* and *mrc1Δ* with the *cdc48-3* allele that is partially defective in CMG disassembly. The indicated diploids (YTM637, YTM638, YTM681 and YTM693) were processed as in Figure 4D.

Figure S4 related to Figure 4

Fission yeast Pof3 and Mcl1 interact via similar domains to budding yeast Dia2 and Ctf4. (**A**) Two-hybrid analysis of full length Mcl1 (1-815) with full length Pof3 (1-577). (B) Analogous assays involving Mcl1 NT (1-321), Mcl1-ΔNT (322-815), Pof3 TPR (1-138) and Pof3-ΔTPR (104-577).

Strain	Genotype
W303-1a	*MAT***a** *ade2-1 ura3-1 his3-1 trp1-1 leu2-3, 112 can1-100*
YTM325	*MAT***a** *MRC1-18MYC (K.l.Trp1) pep4Δ::URA3 ADE2*
YTM326	*MAT***a** *MCM4-5FLAG (hphNT) MRC1-18MYC (K.l.TRP1) pep4Δ::URA3 ADE2*
YASD375	*MAT***a** *TAP-SLD5 (kanMX) pep4Δ::URA3 ADE2*
YTM403	*MAT***a** *ctf4Δ::HIS3 TAP-SLD5 (kanMX) pep4Δ::ADE2*
YLG31	*MAT***a** *mrc1Δ::TRP1 TAP-SLD5 (kanMX) pep4Δ::URA3 ADE2*
YTM265	*MAT***a** *dia2-ΔTPR (hphNT) TAP-SLD5 (kanMX) pep4Δ::URA3 ADE2*
YTM401	*MAT***a** *CDC45-ProteinA (kanMX) pep4Δ::ADE2*
YTM438	*MAT***a** *ctf4Δ::kanMX CDC45-ProteinA (kanMX) pep4Δ::ADE2*
YTM440	*MAT***a** *mrc1Δ::kanMX CDC45-ProteinA (kanMX) pep4Δ::ADE2*
YSS3	*MAT***a** *pep4Δ::ADE2*
YPNK314	*MAT***a** *mrc1Δ::kanMX pep4Δ::ADE2*
YPNK342	*MAT***a** *GAL-MRC1 (K.l.TRP1) pep4Δ::ADE2*
YMM228	*MAT***a** *cdc48-aid (hphNT) ura3-1::ADH1-OsTIR1-9MYC (URA3 & K.l.TRP1) TAP-SLD5 (kanMX) pep4Δ::URA3 ADE2*
YPNK334	*MAT***a** *dia2ΔTPR (hphNT) cdc48-aid (hphNT) ura3-1::ADH1-OsTIR1-9MYC (URA3 & K.l.TRP1) TAP-SLD5 (kanMX) pep4Δ::URA3 ADE2*
YHM130	*MAT***a** *dia2Δ::HIS3 TAP-SLD5 (kanMX) pep4Δ::URA3 ADE2*
YHM28	*MAT***a** *dia2Δ::HIS3*
YMM188	*MAT***a** *cdc48-aid (hphNT)*
YHM306	*MAT***a** *dia2ΔTPR (hphNT)*
YPNK305	*MAT***a** *dia2ΔTPR (hphNT) cdc48-aid (hphNT)*
YTM636	*MAT***a** */ MATα DIA2 / dia2ΔTPR (hphNT) CDC48 / cdc48-3*
YGDP219	*MAT***a** *MCM4-5FLAG (hphNT) MRC1-18MYC (K.l.TRP1) pep4Δ::URA3 ADE2*
YGDP417	*MAT***a** *mec1Δ::ADE2 sml1Δ::HIS3 POL1-6HA (K.l.TRP1) MCM4-5FLAG (hphNT) MRC1-18MYC (K.l.TRP1) pep4Δ::URA3*
YTM179	*MAT***a** *dia2Δ::HIS3 MCM4-5FLAG (hphNT) MRC1-*

	18MYC (K.l.TRP1) pep4Δ::URA3
YTM637	*MAT***a** */ MATα CTF4 / ctf4Δ::HIS3 CDC48 / cdc48-3 ADE2 / ade2-1*
YTM638	*MAT***a** */ MATα MRC1 / mrc1Δ::hphNT CDC48 / cdc48-3*
YTM681	*MAT***a** */ MATα RAD51/ rad51Δ::kanMX CDC48 / cdc48-3*
YTM693	*MAT***a** *5FLAG-dia2-ΔLRR (his3MX) pep4Δ::ADE2*

Supplemental Table S1

Strains used in this study - all based on the W303 yeast genetic background, except for the yeast two-hybrid strain PJ69-4A.

Supplemental Experimental Procedures

Yeast Strains and Growth

The *Saccharomyces cerevisiae* strains that were used in this study are listed in Supplemental Table S1. Cells were grown in rich media (YPD) that contained yeast extract (1%), peptone (2%) and glucose (2%). As required, cells were synchronized in G1 by addition of 7.5 mg/ml alpha-factor mating pheromone and released into S phase by washing twice with fresh YPD media. To inhibit ribonucleotide reductase and slow progression through S-phase, hydroxyurea (HU; Sigma-Aldrich H8627) was added to a final concentration of 200mM. Cells were arrested in G2-phase by addition to the culture medium of 5μg/ml nocodazole (Sigma-Aldrich M1404). To induce degradation of Cdc48-aid, we added 0.5mM 3-indolacetic acid (IAA; I3750, Sigma-Aldrich) to the culture medium for the indicated time.

Yeast Two-Hybrid Assays

We performed two-Hybrid analysis by co-transformation of derivatives of pGADT7 (Gal4 activation domain; *LEU2* marker) and pGBKT7 (Gal4 DNA binding domain; *TRP1* marker) into the yeast strain PJ69-4A. Five independent transformed colonies were mixed together in PBS medium, before spotting ten-fold dilutions from 50,000 to 50 cells onto selective medium. We either used Synthetic Complete medium lacking tryptophan (to select for pGBKT7) and leucine (to select for pGADT7), or else used SC medium lacking tryptophan, leucine and histidine (additionally selective for the two-hybrid interaction).

Immunoprecipitation and immunoblotting of proteins from yeast cell extracts

After harvesting 250ml culture samples (about 2.5 x 10^9 cells), cell extracts were prepared at pH 7.9 or pH 9, in the presence of 100mM or 700mM potassium acetate, as indicated in the figures. Extracts were made as described previously [S1, S2], using a SPEX SamplePrep 6850 Freezer/Mill. In order to digest chromosomal DNA, extracts were incubated for 30 minutes at 4°C with 800 units of benzonase (71206-3, Merck Biosciences). Tagged proteins were isolated by immunoprecipitation with magnetic Dyna-beads M-270 Epoxy (Invitrogen) coupled to rabbit IgG (Sigma S-1265) or M2 anti-FLAG monoclonal antibody (Sigma F3165). Proteins were detected by immunoblotting using polyclonal antibodies previously described [S3], polyclonal anti-FLAG antibody (Sigma F-7425), polyclonal anti-Cdc53 antibody (Santa Cruz y-300, sc-50444), 9E10 anti-MYC antibody (Cancer Research UK) or with Peroxidase:Anti-Peroxidase complex (Sigma P-2026) for TAP-tagged proteins.

Purification of Ctf4 for *in vitro* complementation experiments

Ctf4-TAP was isolated from 2.5 g of frozen yeast as described above, using magnetic beads coupled to rabbit IgG. After a two-hour incubation with yeast extracts at 4˚C, the IgG-coated beads were washed twice with 1 ml of pH 9 wash buffer (100mM Tris-acetate pH 9, 100mM potassium acetate, 10mM magnesium acetate, 0.1% IGEPAL CA-360, 2mM sodium fluoride,

2mM sodium β-glycerophosphate pentahydrate) supplemented with protease inhibitors, once with 1 ml of pH 7.9 wash buffer (100mM HEPES-KOH pH 7.9, 100mM potassium acetate, 10mM magnesium acetate, 0.1% IGEPAL CA-360, 2mM sodium fluoride, 2mM sodium β-glycerophosphate pentahydrate), then once with 1ml of TEV cleavage buffer (100mM HEPES-KOH pH 7.9, 100mM potassium acetate, 10mM magnesium acetate, 0.1% IGEPAL CA-360). The magnetic beads were then agitated for one hour at 24°C in 80μl of TEV cleavage buffer supplemented with 4μl (40U) of AcTEV protease (12575015, Life Technologies). Following elution, the supernatant was removed and 40μl of supernatant was added to each millilitre of the recipient cell extract as indicated (the negative control comprised 40μl of TEV cleavage buffer).

Supplemental References

S1. De Piccoli, G., Katou, Y., Itoh, T., Nakato, R., Shirahige, K., and Labib, K. (2012). Replisome stability at defective DNA replication forks is independent of S phase checkpoint kinases. Molecular cell *45*, 696-704.

S2. Maric, M., Maculins, T., De Piccoli, G., and Labib, K. (2014). Cdc48 and a ubiquitin ligase drive disassembly of the CMG helicase at the end of DNA replication. Science *346*, 1253596.

S3. Gambus, A., Jones, R.C., Sanchez-Diaz, A., Kanemaki, M., van Deursen, F., Edmondson, R.D., and Labib, K. (2006). GINS maintains

association of Cdc45 with MCM in replisome progression complexes at eukaryotic DNA replication forks. Nat Cell Biol *8*, 358-366.
