# Supplementary material for: Tethering of SCFDia2 to the Replisome Promotes Efficient Ubiquitylation and Disassembly of the CMG Helicase
Source: Curr Biol. 2015 Aug 31;25(17):2254–9. doi: 10.1016/j.cub.2015.07.012 (PMC4562905; doi:10.1016/j.cub.2015.07.012)
Supplement: Document S2. Article plus Supplemental Information [file mmc2.pdf]

Current Biology

Report

Tethering of SCF^{Dia2} to the Replisome Promotes Efficient Ubiquitylation and Disassembly of the CMG Helicase

Graphical Abstract

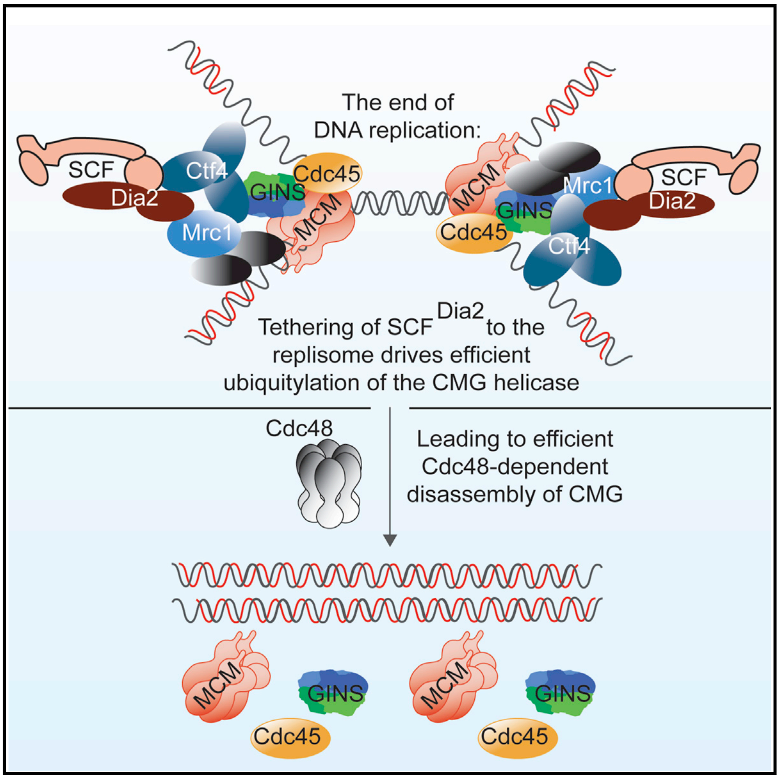

Highlights

- Replisome tethering of SCF^{Dia2} promotes efficient ubiquitylation of the CMG helicase
- Loss of tethering and mutation of Cdc48 cause synthetic CMG disassembly defects

Authors

Timurs Maculins, Pedro Junior Nkosi, Hiroko Nishikawa, Karim Labib

Correspondence

kpmlabib@dundee.ac.uk

In Brief

Disassembly of the CMG helicase is the key regulated step at the end of chromosome replication in eukaryotes, and in budding yeast, this is driven by the ubiquitin ligase SCF^{Dia2} and the Cdc48 segregase. SCF^{Dia2} is tethered to the replisome, and Maculins et al. show that this connection controls the efficiency of CMG ubiquitylation and disassembly.

Maculins et al., 2015, Current Biology *25*, 2254–2259
August 31, 2015 ©2015 The Authors
http://dx.doi.org/10.1016/j.cub.2015.07.012

CellPress
Current Biology
Report

Tethering of SCF^{Dia2} to the Replisome Promotes Efficient Ubiquitylation and Disassembly of the CMG Helicase

Timurs Maculins,[1,3,4] Pedro Junior Nkosi,[2,4] Hiroko Nishikawa,[1] and Karim Labib[2,*]
[1]Cancer Research UK Manchester Institute, University of Manchester, Wilmslow Road, Manchester M20 4BX, UK
[2]MRC Protein Phosphorylation and Ubiquitylation Unit, Sir James Black Centre, College of Life Sciences, University of Dundee, Dow Street, Dundee DD1 5EH, UK
[3]Present address: Postdoctoral Research Programme, Discovery Sciences, Astrazeneca, Alderley Park, Cheshire SK10 4TG, UK
[4]Co-first author
*Correspondence: kpmlabib@dundee.ac.uk
http://dx.doi.org/10.1016/j.cub.2015.07.012
This is an open access article under the CC BY license (http://creativecommons.org/licenses/by/4.0/).

SUMMARY

Disassembly of the Cdc45-MCM-GINS (CMG) DNA helicase, which unwinds the parental DNA duplex at eukaryotic replication forks, is the key regulated step during replication termination but is poorly understood [1, 2]. In budding yeast, the F-box protein Dia2 drives ubiquitylation of the CMG helicase at the end of replication, leading to a disassembly pathway that requires the Cdc48 segregase [3]. The substrate-binding domain of Dia2 comprises leucine-rich repeats, but Dia2 also has a TPR domain at its amino terminus that interacts with the Ctf4 and Mrc1 subunits of the replisome progression complex [4, 5], which assembles around the CMG helicase at replication forks [6]. Previous studies suggested two disparate roles for the TPR domain of Dia2, either mediating replisome-specific degradation of Mrc1 and Ctf4 [4] or else tethering SCF^{Dia2} (SCF [Skp1/cullin/F-box protein]) to the replisome to increase its local concentration at replication forks [5]. Here, we show that SCF^{Dia2} does not mediate replisome-specific degradation of Mrc1 and Ctf4, either during normal S phase or in response to replication stress. Instead, the tethering of SCF^{Dia2} to the replisome progression complex increases the efficiency of ubiquitylation of the Mcm7 subunit of CMG, both in vitro and in vivo. Correspondingly, loss of tethering reduces the efficiency of CMG disassembly in vivo and is synthetic lethal in combination with a disassembly-defective allele of *CDC48*. Residual ubiquitylation of Mcm7 in *dia2-ΔTPR* cells is still CMG specific, highlighting the complex regulation of the final stages of chromosome replication, about which much still remains to be learned.

RESULTS AND DISCUSSION

CMG Disassembly Explains the Apparent Instability of Replisome-Associated Mrc1 and Ctf4

Previous work showed that the association of budding yeast Mrc1 and Ctf4 with the Cdc45-MCM-GINS (CMG) helicase was lost in control cells, but not in *dia2Δ*, when cycloheximide was used to inhibit protein synthesis in asynchronous cell cultures [4]. This was taken as evidence that SCF^{Dia2} specifically ubiquitylates the fraction of Mrc1 and Ctf4 that is incorporated into the replisome progression complex at replication forks. We repeated the same experiment with control cells expressing *DIA2* by immunoprecipitating the Mcm4 helicase subunit from cell extracts after addition of cycloheximide. Whereas Mcm4 still associated with the remaining subunits of the Mcm2-7 complex in cycloheximide-treated cells, association with all other RPC subunits was lost (Figure 1A). Rather than reflecting the specific degradation of RPC-associated Mrc1 and Ctf4, these data thus indicated that the RPC is no longer present when control cells are treated with cycloheximide. A simple explanation for this is provided by the fact that protein synthesis is required for G1 phase cells to enter S phase, but S phase cells can complete DNA replication without ongoing protein synthesis [7–9]. Consistent with this view, flow cytometry data from the same experiment indicated that the S phase population of cells was lost upon addition of cycloheximide to the asynchronous cell culture (Figure 1Bi). Cycloheximide should thus block the assembly, but not the disassembly, of the RPC.

To confirm that loss of the RPC in cycloheximide-treated cells reflects CMG disassembly during the completion of chromosome replication, we arrested cells with hydroxyurea in early S phase, prior to cycloheximide treatment. In contrast to the above experiment, the association of Mcm4 with all other RPC components including Ctf4 and Mrc1 was preserved in hydroxyurea-arrested cells upon treatment with cycloheximide (Figure 1C), reflecting the stable persistence of the replisome at stalled replication forks. Moreover, the same was true when cells lacking the Mec1 checkpoint kinase were arrested with hydroxyurea and then treated with cycloheximide, indicating

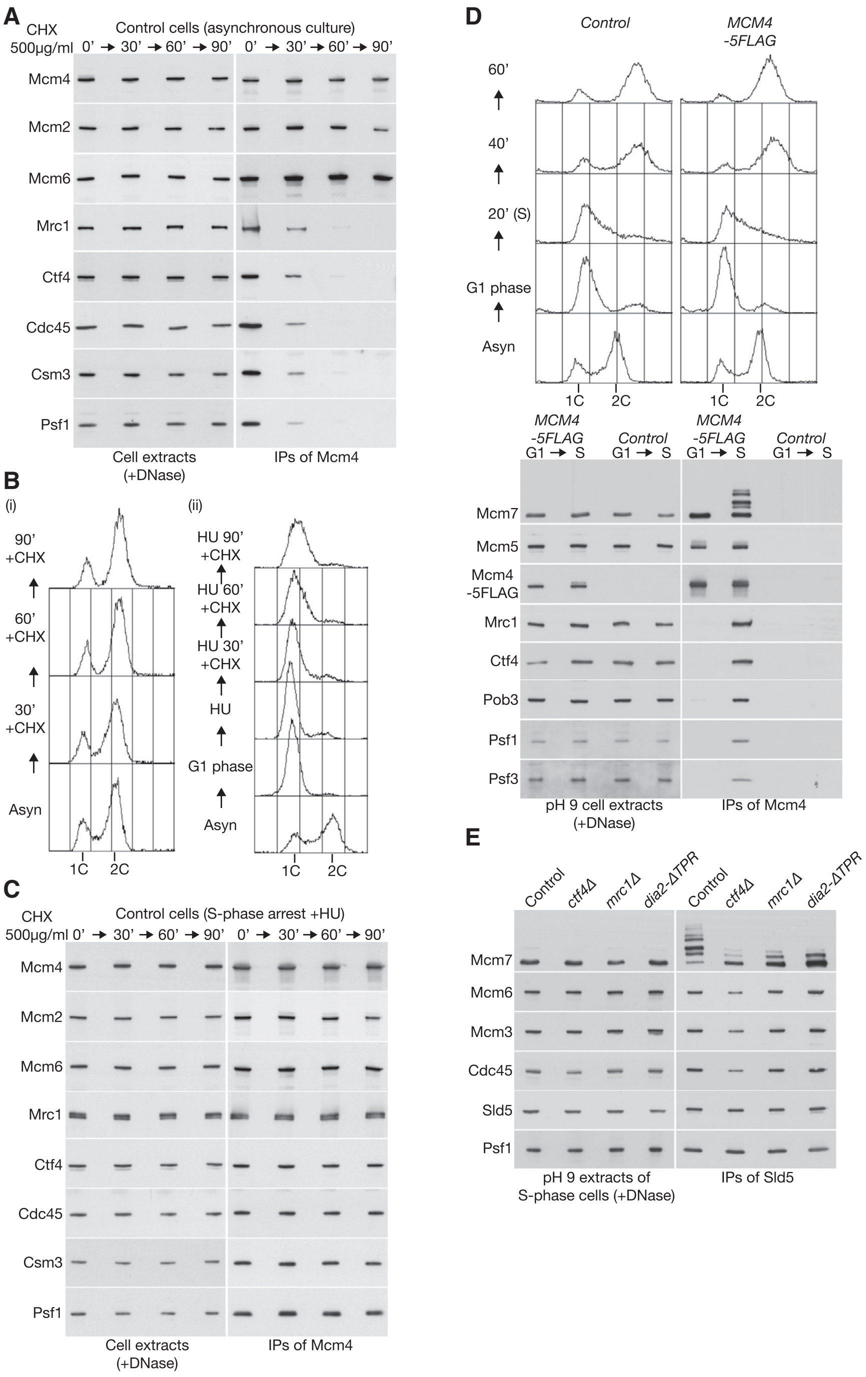

Figure 1. Tethering of SCF^{Dia2} to the Replisome Progression Complex Increases the Efficiency of CMG Ubiquitylation In Vitro

(A) An asynchronous culture of *MCM4-5FLAG MRC1-18MYC* cells (YGDP219) was grown at 30°C, before addition of 500 μg/ml cycloheximide for the indicated times. Cell extracts were treated with DNase before immunoprecipitation of Mcm4-5FLAG and detection of the indicated proteins by immunoblotting.

(B) (i) Flow cytometry analysis from the same experiment. (ii) The same strain as above was arrested in G1 phase and then released into S phase for 60 min in the presence of 0.2 M hydroxyurea. Cycloheximide was added for the indicated times and samples processed as before.

(C) The samples from (Bii) were processed as in (A).

(D) Control cells (YTM325) and *MCM4-5FLAG* (YTM326) were synchronized at 30°C in the G1 phase of the cell cycle by addition of mating pheromone, before release into S phase for 20 min. DNA content was monitored by flow cytometry (upper panels). "pH 9 cell extracts" were then prepared as described in the Supplemental Experimental Procedures and incubated with magnetic beads coupled to anti-FLAG monoclonal antibody. The immunoprecipitated proteins were then monitored by immunoblotting (lower panels).

(E) Control (YASD375), *ctf4Δ* (YTM403), *mrc1Δ* (YLG31), and *dia2-ΔTPR* (YTM265) were synchronized in early S phase as above, before immunoprecipitation of TAP-Sld5 from pH 9 cell extracts on IgG beads.

See also Figures S1 and S2.

that the persistent association of Mrc1 and Ctf4 with the RPC in hydroxyurea-arrested control cells did not reflect the inhibition of SCF^{Dia2} by the S phase checkpoint pathway (Figure S1A).

Subsequently, we treated an asynchronous culture of *dia2Δ* cells with cycloheximide and observed the persistent association of Mcm4 with all tested RPC components including Mrc1 and Ctf4 (Figure S1B). These data are explicable by the failure of *dia2Δ* cells to disassemble the CMG helicase at the end of S phase [3].

Finally, we directly examined RPC ubiquitylation in an extract of S phase yeast cells, using conditions that we had previously shown to support efficient in vitro ubiquitylation of CMG on its Mcm7 subunit, dependent upon SCF^{Dia2} and the Cdc34 ubiquitin-conjugating enzyme [3]. Whereas the in vitro ubiquitylation of CMG was easily detected in these "pH 9 cell extracts," we did not detect ubiquitylation of the associated RPC subunits including Mrc1 and Ctf4 (Figure 1D). Taken together, the preceding experiments reflect the disassembly of the CMG helicase during replication termination in control cells and the failure of CMG disassembly in *dia2Δ* cells but do not provide evidence for the replisome-specific ubiquitylation of Mrc1 and Ctf4 by SCF^{Dia2}.

Tethering of SCF^{Dia2} to the Replisome Progression Complex Increases the Efficiency of CMG Ubiquitylation In Vitro

To examine whether ubiquitylation of the CMG helicase is dependent upon tethering of SCF^{Dia2} to the replisome progression complex (Figure S2), we compared the ability of S phase extracts of control cells *ctf4Δ*, *mrc1Δ*, or *dia2-ΔTPR* to support in vitro

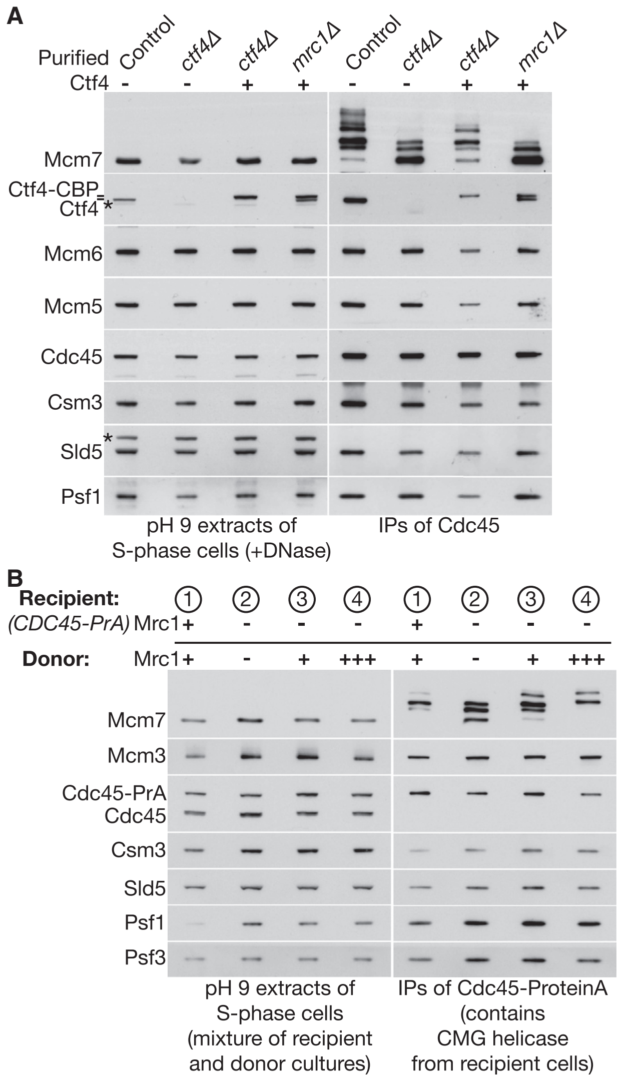

Figure 2. The CMG Ubiquitylation Defects of *ctf4Δ* and *mrc1Δ* Can Be Rescued In Vitro

(A) S phase cell extracts of control (YTM401), *ctf4Δ* (YTM438), and *mrc1Δ* (YTM440) were prepared at pH 9 as above and complemented with buffer or purified Ctf4 as indicated, before immunoprecipitation of Cdc45-ProteinA. The indicated proteins were then monitored by immunoblotting. Asterisks denote non-specific bands.

(B) To test for in vitro rescue of the ubiquitylation defect of *mrc1Δ* cell extracts, we synchronized the indicated *CDC45-ProteinA* "recipient strains" (1, YTM401; 2–4, YTM440) and *CDC45* "donor strains" (1–4, YSS3, YPNK314, YSS3, and YPNK342, respectively) in S phase at 30°C. Each of the indicated pairs of recipient and donor cultures were then mixed and used to prepare a single cell extract at pH 9 as above. After digestion of chromosomal DNA, the CMG helicase from recipient cells was isolated by immunoprecipitation of its ProteinA-tagged-Cdc45 subunit.

CMG ubiquitylation. As seen in our previous study [3], ubiquitylation of Mcm7 was restricted to the specific fraction that is present in the CMG helicase, which we isolated by immunoprecipitation of the Sld5 subunit of GINS (Figure 1E). In control cell extracts, almost all CMG complexes had ubiquitylated Mcm7 under these conditions, producing a ladder of modified Mcm7 bands in which unmodified Mcm7 was only a minor form (Figure 1E; IPs of Sld5; control). Although ubiquitylated Mcm7 could still be detected when CMG was isolated from extracts of *ctf4Δ*, *mrc1Δ*, or *dia2-ΔTPR*, ubiquitylation was much reduced in all three cases compared to the control (Figure 1E). These findings indicated that the tethering of SCF^{Dia2} to the RPC, by interaction of the TPR of Dia2 with Mrc1 and Ctf4, is important for the efficiency of CMG ubiquitylation of CMG in vitro.

To confirm that loss of tethering reduced the capacity of SCF^{Dia2} to drive in vitro ubiquitylation of the CMG helicase, we repeated the above experiment with S phase extracts of control, *ctf4Δ*, or *mrc1Δ* and then complemented the extracts with buffer or with purified Ctf4 protein. Critically, addition of purified Ctf4 to the *ctf4Δ* extract restored the efficiency of ubiquitylation, producing a very similar pattern to the control extract (with di-ubiquitylated Mcm7 being the predominant form in the isolated CMG material), whereas addition of Ctf4 to an extract of *mrc1Δ* cells had no effect (Figure 2A). Similarly, we showed that the CMG ubiquitylation defect of *mrc1Δ* extracts could be rescued in vitro by mixing with extracts of cells that expressed Mrc1. We synchronized "recipient" cultures of *CDC45-ProteinA* in S phase alongside "donor cultures" expressing untagged *CDC45* and then mixed cultures as indicated in Figure 2B, before making cell extracts and isolating "'recipient CMG" by immunoprecipitation of Cdc45-ProteinA. A donor extract expressing Mrc1 was able to rescue the in vitro ubiquitylation defect of an *mrc1Δ* recipient extract (Figure 2B, sample 3), whereas an extract overexpressing Mrc1 further enhanced the ubiquitylation of CMG (Figure 2B, sample 4). These findings demonstrate that the TPR-dependent tethering of SCF^{Dia2} to the RPC serves to increase the efficiency of CMG ubiquitylation in vitro.

Tethering of SCF^{Dia2} to the Replisome Progression Complex Is Important for Efficient CMG Ubiquitylation In Vivo

Ubiquitylation of the CMG helicase is restricted to the end of chromosome replication in vivo, when it is coupled rapidly to Cdc48-dependent disassembly [3]. For visualization of ubiquitylated CMG in vivo, it is necessary to inactivate Cdc48 before cells terminate DNA replication and then prepare "high salt" extracts that block the in vitro ubiquitylation of the CMG helicase. In order to assess the contribution of replisome tethering of SCF^{Dia2} to CMG ubiquitylation in vivo, we synchronized *cdc48-aid* and *dia2-ΔTPR cdc48-aid* cells (aid [auxin inducible degron]) in early S phase and then depleted Cdc48-aid, before allowing cells to proceed with chromosome replication (Figure 3A). As shown in Figure 3B, in vivo ubiquitylation of the Mcm7 subunit of CMG could still be detected at the end of S phase in *dia2-ΔTPR cdc48-aid* cells but was markedly reduced. These data indicate that the tethering of SCF^{Dia2} to the RPC increases the efficiency of CMG ubiquitylation at the end of chromosome replication in budding yeast.

Impaired Ubiquitylation of CMG in *dia2-ΔTPR* Cells Produces a Defect in CMG Disassembly

Cells lacking Dia2 have a very high rate of genome instability, are unable to grow at low temperatures, and are sensitive to DNA-damaging agents that perturb the progression of DNA replication forks [5, 10–12]. Dia2 drives the disassembly of the CMG helicase at the end of chromosome replication so that the absence of Dia2 causes CMG to persist into G1 phase of

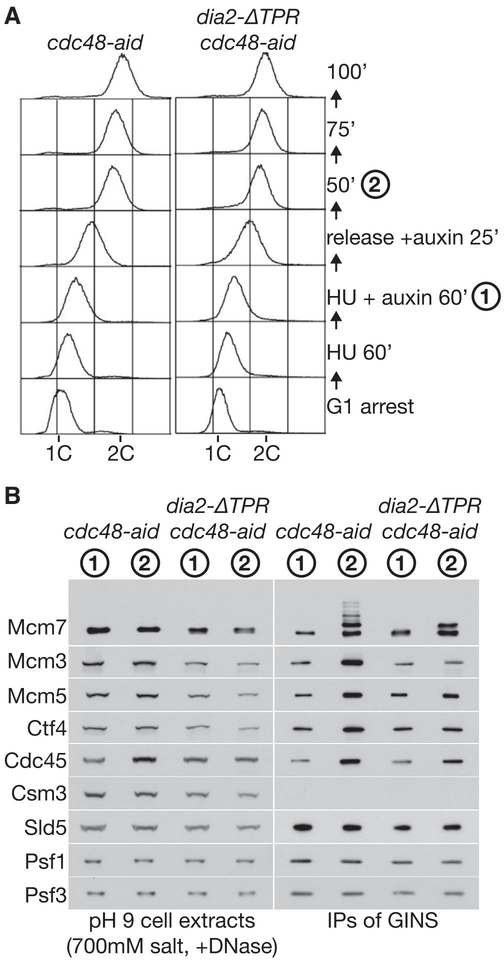

Figure 3. Tethering of SCF^{Dia2} to the Replisome Progression Complex Increases the Efficiency of In Vivo CMG Ubiquitylation at the End of S Phase

(A) *cdc48-aid* (YMM228) and *cdc48-aid dia2-ΔTPR* (YPNK334) were synchronized in G1 phase at 30°C and then released into S phase for 60 min in the presence of 0.2 M hydroxyurea. For depletion of Cdc48-aid, 0.5 mM auxin was added for 60 min, before release into fresh medium containing auxin but lacking hydroxyurea. DNA content was monitored by flow cytometry, and samples were taken at the indicated times ("1" and "2") to prepare pH 9 cell extracts containing 700 mM salt.

(B) CMG helicase was isolated as above by immunoprecipitation of TAP-tagged Sld5 subunit.

the next cell cycle [3], but at present, it is not known how this defect is linked to the other phenotypes of *dia2Δ* cells.

Although CMG was not detected during G1 phase in *dia2-ΔTPR* cells grown at 30°C, or in *cdc48-aid* cells grown at 30°C in medium lacking auxin (permissive conditions, in which the phenotype just reflects the C-terminal tag on Cdc48), we found that the combination of *dia2-ΔTPR* with *cdc48-aid* produced a synthetic defect in CMG disassembly that resembled the phenotype of *dia2Δ* cells at 30°C (Figure 4A). Moreover, the *dia2-ΔTPR cdc48-aid* strain also shared the sensitivity of *dia2Δ* cells to the DNA-damaging agent methyl methanesulfonate (Figure 4B; note that cells were grown in the absence of auxin). These findings suggested that *dia2-ΔTPR* cells have a partial defect in CMG disassembly, even though helicase disassembly is still completed by the end of the cell cycle. Accordingly, we found that asynchronous cultures of *dia2-ΔTPR* contained slightly more CMG helicase than control cells (Figure 4C; we cannot exclude that the activation of more origins during S phase might also contribute to this effect in *dia2-ΔTPR* cells).

We previously showed that the *cdc48-3* allele has a partial defect in CMG disassembly at the permissive temperature of 24°C [3]. Strikingly, we found that *dia2-ΔTPR* was synthetic lethal with *cdc48-3* at 24°C (Figure 4D), reminiscent of the cold-sensitive phenotype of *dia2Δ* cells [5]. Moreover, *ctf4Δ* was also synthetic lethal with *cdc48-3* at 24°C, whereas *mrc1Δ cdc48-3* showed a synthetic growth defect (Figure S3). In contrast, deletion of factors with other roles at defective replication forks, such as Top3 or Rad51, did not cause synthetic lethality with *cdc48-3* (Figure S3). Taken together, these findings indicate that tethering of SCF^{Dia2} to the RPC contributes to efficient disassembly of the CMG helicase at the end of chromosome replication in budding yeast.

CMG disassembly represents the key regulated step during replication termination, which drives replisome disassembly and must not occur prematurely [1, 2]. Although CMG ubiquitylation and Cdc48-dependent disassembly have been conserved from budding yeast to vertebrates, the mechanism and regulation of CMG disassembly are still very poorly characterized in all eukaryotes. Budding yeast SCF^{Dia2} is currently the only ubiquitin ligase that has been shown to drive CMG disassembly in any species and thus provides an important model system with which to study the underlying principles.

It seems likely that ubiquitylation is rate limiting for CMG disassembly, although this remains to be demonstrated by mapping and mutation of the ubiquitylation sites in Mcm7. It is clear that Mcm7 ubiquitylation is regulated in an exquisite fashion on many levels, both spatially and temporally. One key aspect is that ubiquitylation of Mcm7 only occurs in the context of the CMG helicase and thus is restricted to replication forks. Our findings in this study indicate that SCF^{Dia2} is preferentially targeted to the replisome progression complex, rather than simply to the CMG helicase itself. Tethering of SCF^{Dia2} to the RPC increases the efficiency of CMG ubiquitylation and involves the interaction of the TPR domain of Dia2 with both Ctf4 and Mrc1, which only come together in the context of the RPC. Nevertheless, the residual ubiquitylation of Mcm7 in cells that cannot tether SCF^{Dia2} to the RPC is also CMG specific (Figures 2 and 3). One possibility is that the leucine-rich repeats of Dia2 target the ligase to Mcm7 in a CMG-dependent manner that requires a structural change in the helicase during termination.

Factors that drive the assembly of the CMG helicase during the initiation of replication, such as the Cdc7 kinase or the TopBP1 adaptor protein, are currently being pursued as targets for new anti-cancer therapies in tumors that retain inherent defects in chromosome replication [13–15]. It will be interesting to explore the potential of CMG disassembly for future therapies, and it will thus be important to determine the ubiquitin ligase(s) driving CMG disassembly in human cells and other eukaryotic species. Orthologs of Dia2 are present in other yeasts [16], including fission yeast Pof3 that appears to use its TPR domain to target Ctf4 in a manner analogous to budding yeast Dia2 (Figure S4). Moreover, a small-molecule inhibitor of cullin neddylation blocks

CellPress

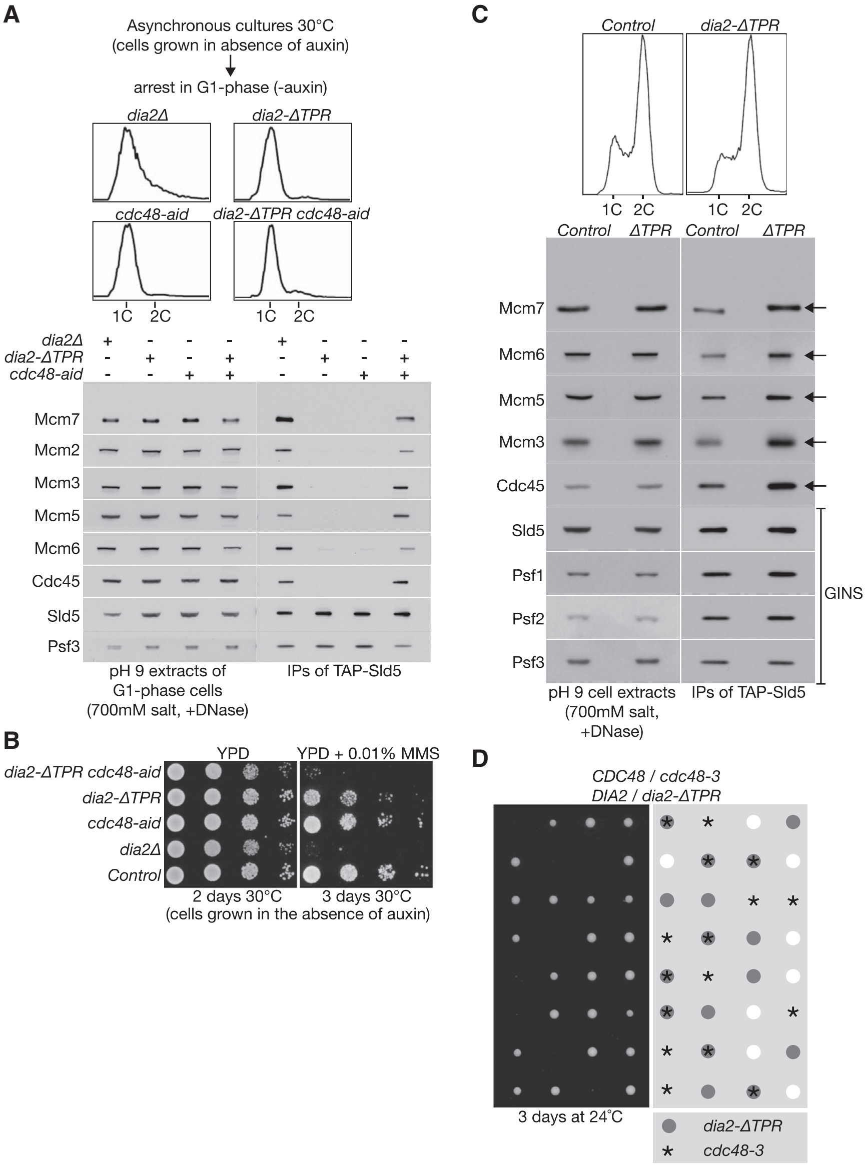

Figure 4. The CMG Disassembly Defect of *dia2-ΔTPR* Is Augmented by Mutations in *CDC48*

(A) *dia2Δ* (YHM130), *dia2-ΔTPR* (YTM265), *cdc48-aid* (YMM228), and *dia2-ΔTPR cdc48-aid* (YPNK334) were arrested in G1 phase at 30°C, in medium lacking auxin (permissive conditions for *cdc48-aid*), before isolation of TAP-Sld5 from "high-salt pH 9 cell extracts."

(B) Serial dilutions of the indicated strains were grown on rich medium (YPD) in the absence or presence of the DNA-damaging agent methyl methanesulfonate.

(C) Asynchronous cultures of control (YASD375) and *dia2-ΔTPR* (YTM265) were grown at 30°C, before isolation of TAP-Sld5 from high-salt pH 9 cell extracts as above. The arrows denote the increased association of Cdc45 and Mcm2-7 proteins with GINS in extracts of asynchronous *dia2-ΔTPR* cells.

(D) The diploid strain *CDC48/cdc48-3 DIA2/dia2-ΔTPR* was sporulated and subjected to tetrad analysis. The germinated spores were grown for 3 days at 24°C before imaging.

See also Figures S3 and S4.

CMG ubiquitylation at the end of DNA replication in frog egg extracts [17]. Nevertheless, homologs of Dia2 have yet to be identified in higher eukaryotes, and it is possible that an unrelated E3 ligase ubiquitylates CMG at the end of chromosome replication in other species. Functional screens for factors driving CMG disassembly in higher eukaryotes will be an important challenge for future studies.

SUPPLEMENTAL INFORMATION

Supplemental Information includes Supplemental Experimental Procedures, four figures, and one table and can be found with this article online at http://dx.doi.org/10.1016/j.cub.2015.07.012.

AUTHOR CONTRIBUTIONS

T.M. performed the experiments in Figures 1, 2A, S1, and S3. P.J.N. carried out the experiments in Figures 2B, 3, 4, and S2. H.N. performed the experiments in Figure S4. K.L. designed the project in collaboration with T.M., P.J.N., and H.N. and wrote the manuscript.

ACKNOWLEDGMENTS

We gratefully acknowledge the support of the Medical Research Council, the Wellcome Trust (references 097945/B/11/Z for flow cytometry and for 102943/Z/13/Z for award to K.L.), and Cancer Research UK for funding our work. We thank Takashi Toda for providing plasmids encoding fission yeast Pof3 and Mcl1.

Received: May 6, 2015
Revised: June 30, 2015
Accepted: July 2, 2015
Published: August 6, 2015

REFERENCES

1. Bell, S.P. (2014). DNA replication. Terminating the replisome. Science *346*, 418–419.

2. Lengronne, A., and Pasero, P. (2014). Closing the MCM cycle at replication termination sites. EMBO Rep. *15*, 1226–1227.
3. Maric, M., Maculins, T., De Piccoli, G., and Labib, K. (2014). Cdc48 and a ubiquitin ligase drive disassembly of the CMG helicase at the end of DNA replication. Science *346*, 1253596.
4. Mimura, S., Komata, M., Kishi, T., Shirahige, K., and Kamura, T. (2009). SCF(Dia2) regulates DNA replication forks during S-phase in budding yeast. EMBO J. *28*, 3693–3705.
5. Morohashi, H., Maculins, T., and Labib, K. (2009). The amino-terminal TPR domain of Dia2 tethers SCF(Dia2) to the replisome progression complex. Curr. Biol. *19*, 1943–1949.
6. Gambus, A., Jones, R.C., Sanchez-Diaz, A., Kanemaki, M., van Deursen, F., Edmondson, R.D., and Labib, K. (2006). GINS maintains association of Cdc45 with MCM in replisome progression complexes at eukaryotic DNA replication forks. Nat. Cell Biol. *8*, 358–366.
7. Hereford, L.M., and Hartwell, L.H. (1973). Role of protein synthesis in the replication of yeast DNA. Nat. New Biol. *244*, 129–131.
8. Hereford, L.M., and Hartwell, L.H. (1974). Sequential gene function in the initiation of *Saccharomyces cerevisiae* DNA synthesis. J. Mol. Biol. *84*, 445–461.
9. Tercero, J.A., Longhese, M.P., and Diffley, J.F. (2003). A central role for DNA replication forks in checkpoint activation and response. Mol. Cell *11*, 1323–1336.
10. Blake, D., Luke, B., Kanellis, P., Jorgensen, P., Goh, T., Penfold, S., Breitkreutz, B.J., Durocher, D., Peter, M., and Tyers, M. (2006). The F-box protein Dia2 overcomes replication impedance to promote genome stability in Saccharomyces cerevisiae. Genetics *174*, 1709–1727.
11. Koepp, D.M., Kile, A.C., Swaminathan, S., and Rodriguez-Rivera, V. (2006). The F-box protein Dia2 regulates DNA replication. Mol. Biol. Cell *17*, 1540–1548.
12. Pan, X., Ye, P., Yuan, D.S., Wang, X., Bader, J.S., and Boeke, J.D. (2006). A DNA integrity network in the yeast Saccharomyces cerevisiae. Cell *124*, 1069–1081.
13. Chowdhury, P., Lin, G.E., Liu, K., Song, Y., Lin, F.T., and Lin, W.C. (2014). Targeting TopBP1 at a convergent point of multiple oncogenic pathways for cancer therapy. Nat. Commun. *5*, 5476.
14. Montagnoli, A., Moll, J., and Colotta, F. (2010). Targeting cell division cycle 7 kinase: a new approach for cancer therapy. Clin. Cancer Res. *16*, 4503–4508.
15. Rodriguez-Acebes, S., Proctor, I., Loddo, M., Wollenschlaeger, A., Rashid, M., Falzon, M., Prevost, A.T., Sainsbury, R., Stoeber, K., and Williams, G.H. (2010). Targeting DNA replication before it starts: Cdc7 as a therapeutic target in p53-mutant breast cancers. Am. J. Pathol. *177*, 2034–2045.
16. Katayama, S., Kitamura, K., Lehmann, A., Nikaido, O., and Toda, T. (2002). Fission yeast F-box protein Pof3 is required for genome integrity and telomere function. Mol. Biol. Cell *13*, 211–224.
17. Moreno, S.P., Bailey, R., Campion, N., Herron, S., and Gambus, A. (2014). Polyubiquitylation drives replisome disassembly at the termination of DNA replication. Science *346*, 477–481.

Current Biology

Supplemental Information

Tethering of SCF^{Dia2} to the Replisome Promotes Efficient Ubiquitylation and Disassembly of the CMG Helicase

Timurs Maculins, Pedro Junior Nkosi, Hiroko Nishikawa, and Karim Labib

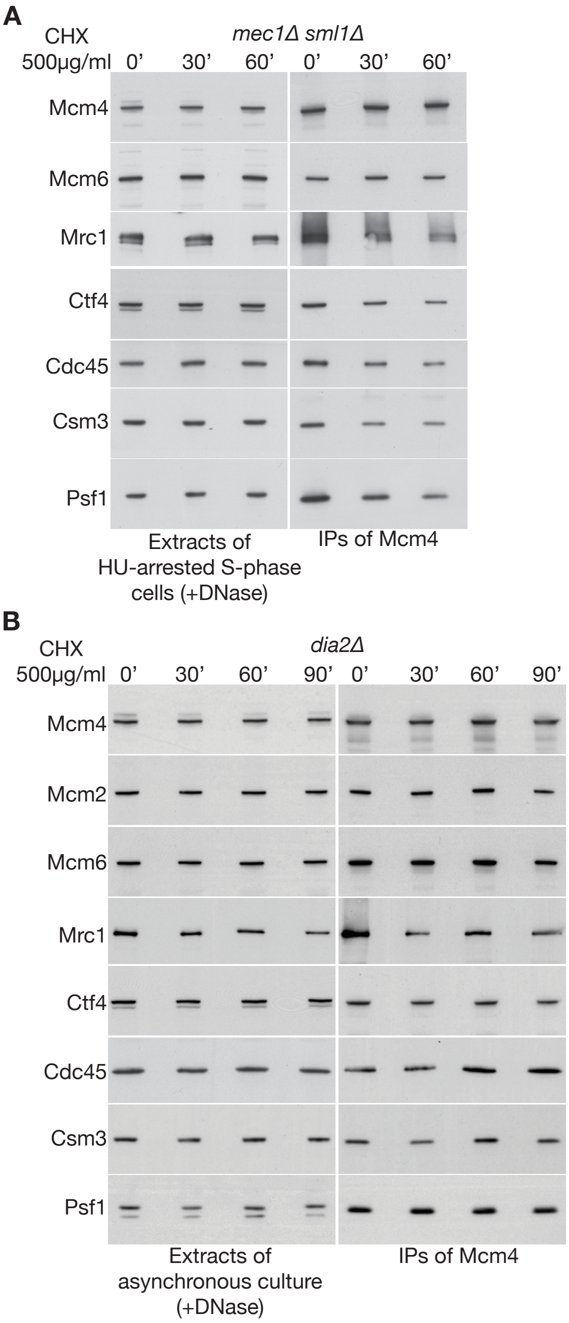
A
CHX
500µg/ml
mec1Δ sml1Δ
0'
30'
60'
0'
30'
60'
Mcm4
Mcm6
Mrc1
Ctf4
Cdc45
Csm3
Psf1
Extracts of HU-arrested S-phase cells (+DNase)
IPs of Mcm4
B
CHX
500µg/ml
dia2Δ
0'
30'
60'
90'
0'
30'
60'
90'
Mcm4
Mcm2
Mcm6
Mrc1
Ctf4
Cdc45
Csm3
Psf1
Extracts of asynchronous culture (+DNase)
IPs of Mcm4

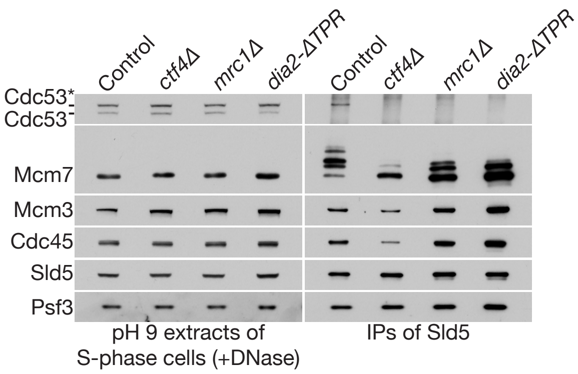
Control
ctf4Δ
mrc1Δ
dia2-ΔTPR
Control
ctf4Δ
mrc1Δ
dia2-ΔTPR
Cdc53*
Cdc53
Mcm7
Mcm3
Cdc45
Sld5
Psf3
pH 9 extracts of
S-phase cells (+DNase)
IPs of Sld5

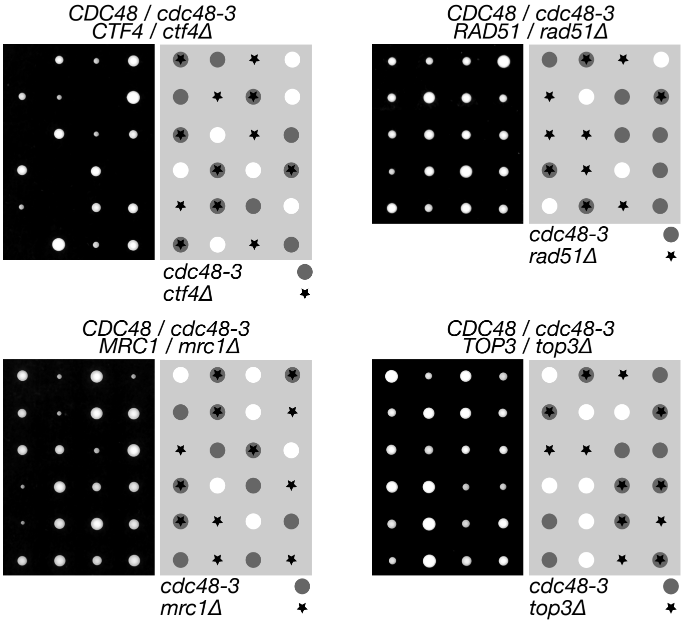
CDC48 / cdc48-3
CTF4 / ctf4Δ
cdc48-3
ctf4Δ
CDC48 / cdc48-3
RAD51 / rad51Δ
cdc48-3
rad51Δ
CDC48 / cdc48-3
MRC1 / mrc1Δ
cdc48-3
mrc1Δ
CDC48 / cdc48-3
TOP3 / top3Δ
cdc48-3
top3Δ

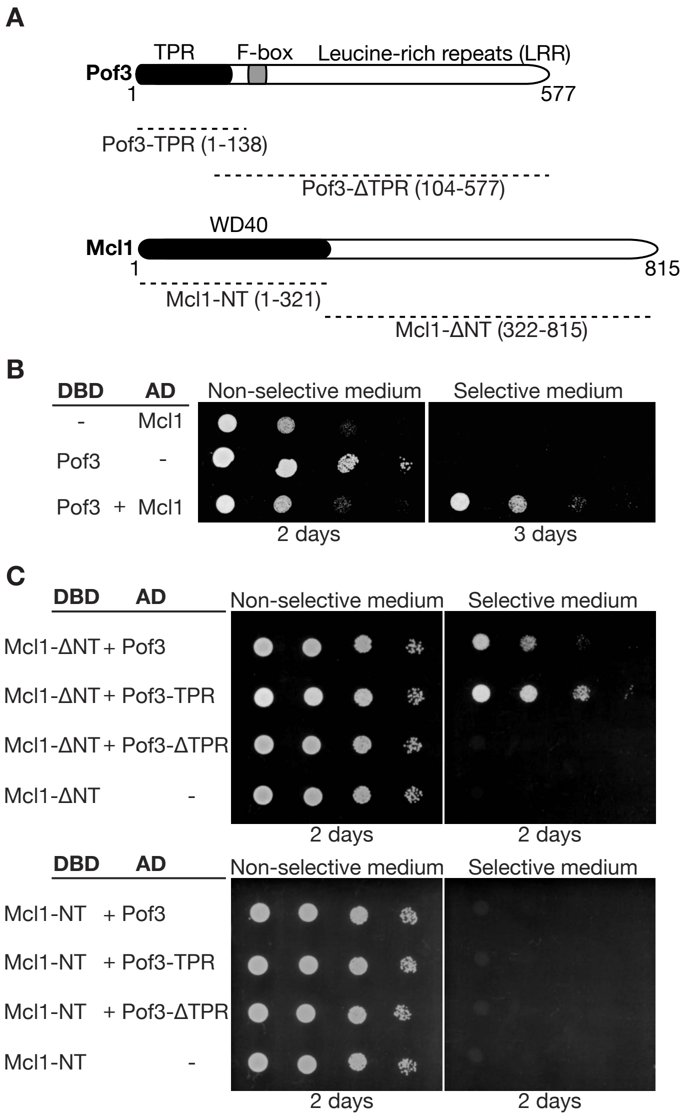
A
TPR
F-box
Leucine-rich repeats (LRR)
Pof3
1
577
Pof3-TPR (1-138)
Pof3-ΔTPR (104-577)
WD40
Mcl1
1
815
Mcl1-NT (1-321)
Mcl1-ΔNT (322-815)
B
DBD
AD
Non-selective medium
Selective medium
- Mcl1
Pof3 -
Pof3 + Mcl1
2 days
3 days
C
DBD
AD
Non-selective medium
Selective medium
Mcl1-ΔNT + Pof3
Mcl1-ΔNT + Pof3-TPR
Mcl1-ΔNT + Pof3-ΔTPR
Mcl1-ΔNT -
2 days
2 days
DBD
AD
Non-selective medium
Selective medium
Mcl1-NT + Pof3
Mcl1-NT + Pof3-TPR
Mcl1-NT + Pof3-ΔTPR
Mcl1-NT -
2 days
2 days

Figure S1 related to Figure 1

Mrc1 and Ctf4 still associate with the replisome when CMG disassembly cannot occur. (**A**) A *mec1Δ sml1Δ* strain (YGDP417) was arrested in G1-phase at 30°C and then released into S-phase in the presence of 0.2M hydroxyurea for 60', before addition of cycloheximide for the indicated times. Samples were then processed as above. (**B**) An asynchronous culture of *dia2Δ* cells (YTM179) was processed as in Figure S1A above.

Figure S2 related to Figure 1

Tethering of SCF^{Dia2} to the replisome is broken by mutation of Ctf4, Mrc1, or the TPR domain of Dia2. The experiment in Figure 1E was repeated, and the association of SCF^{Dia2} with the CMG helicase monitored by immunoblotting of the Cdc53 cullin subunit. Note that Cdc53 is present in two forms in cell extracts: a faster migrating form that represents unmodified Cdc53, and a slower migrating form corresponding to neddylated Cdc53 (marked Cdc53*). The latter associates preferentially with the CMG helicase. The association of SCF^{Dia2} with the replisome is dependent upon Ctf4, Mrc1 and the TPR domain of Dia2.

Figure S3 related to Figure 4

Synthetic interactions of *ctf4Δ* and *mrc1Δ* with the *cdc48-3* allele that is partially defective in CMG disassembly. The indicated diploids (YTM637, YTM638, YTM681 and YTM693) were processed as in Figure 4D.

Figure S4 related to Figure 4

Fission yeast Pof3 and Mcl1 interact via similar domains to budding yeast Dia2 and Ctf4. (**A**) Two-hybrid analysis of full length Mcl1 (1-815) with full length Pof3 (1-577). (B) Analogous assays involving Mcl1 NT (1-321), Mcl1-ΔNT (322-815), Pof3 TPR (1-138) and Pof3-ΔTPR (104-577).

Strain	Genotype
W303-1a	*MAT***a** *ade2-1 ura3-1 his3-1 trp1-1 leu2-3, 112 can1-100*
YTM325	*MAT***a** *MRC1-18MYC (K.l.Trp1) pep4Δ::URA3 ADE2*
YTM326	*MAT***a** *MCM4-5FLAG (hphNT) MRC1-18MYC (K.l.TRP1) pep4Δ::URA3 ADE2*
YASD375	*MAT***a** *TAP-SLD5 (kanMX) pep4Δ::URA3 ADE2*
YTM403	*MAT***a** *ctf4Δ::HIS3 TAP-SLD5 (kanMX) pep4Δ::ADE2*
YLG31	*MAT***a** *mrc1Δ::TRP1 TAP-SLD5 (kanMX) pep4Δ::URA3 ADE2*
YTM265	*MAT***a** *dia2-ΔTPR (hphNT) TAP-SLD5 (kanMX) pep4Δ::URA3 ADE2*
YTM401	*MAT***a** *CDC45-ProteinA (kanMX) pep4Δ::ADE2*
YTM438	*MAT***a** *ctf4Δ::kanMX CDC45-ProteinA (kanMX) pep4Δ::ADE2*
YTM440	*MAT***a** *mrc1Δ::kanMX CDC45-ProteinA (kanMX) pep4Δ::ADE2*
YSS3	*MAT***a** *pep4Δ::ADE2*
YPNK314	*MAT***a** *mrc1Δ::kanMX pep4Δ::ADE2*
YPNK342	*MAT***a** *GAL-MRC1 (K.l.TRP1) pep4Δ::ADE2*
YMM228	*MAT***a** *cdc48-aid (hphNT) ura3-1::ADH1-OsTIR1-9MYC (URA3 & K.l.TRP1) TAP-SLD5 (kanMX) pep4Δ::URA3 ADE2*
YPNK334	*MAT***a** *dia2ΔTPR (hphNT) cdc48-aid (hphNT) ura3-1::ADH1-OsTIR1-9MYC (URA3 & K.l.TRP1) TAP-SLD5 (kanMX) pep4Δ::URA3 ADE2*
YHM130	*MAT***a** *dia2Δ::HIS3 TAP-SLD5 (kanMX) pep4Δ::URA3 ADE2*
YHM28	*MAT***a** *dia2Δ::HIS3*
YMM188	*MAT***a** *cdc48-aid (hphNT)*
YHM306	*MAT***a** *dia2ΔTPR (hphNT)*
YPNK305	*MAT***a** *dia2ΔTPR (hphNT) cdc48-aid (hphNT)*
YTM636	*MAT***a** */ MATα DIA2 / dia2ΔTPR (hphNT) CDC48 / cdc48-3*
YGDP219	*MAT***a** *MCM4-5FLAG (hphNT) MRC1-18MYC (K.l.TRP1) pep4Δ::URA3 ADE2*
YGDP417	*MAT***a** *mec1Δ::ADE2 sml1Δ::HIS3 POL1-6HA (K.l.TRP1) MCM4-5FLAG (hphNT) MRC1-18MYC (K.l.TRP1) pep4Δ::URA3*
YTM179	*MAT***a** *dia2Δ::HIS3 MCM4-5FLAG (hphNT) MRC1-*

	18MYC (K.l.TRP1) pep4Δ::URA3
YTM637	*MAT***a** */ MATα CTF4 / ctf4Δ::HIS3 CDC48 / cdc48-3 ADE2 / ade2-1*
YTM638	*MAT***a** */ MATα MRC1 / mrc1Δ::hphNT CDC48 / cdc48-3*
YTM681	*MAT***a** */ MATα RAD51/ rad51Δ::kanMX CDC48 / cdc48-3*
YTM693	*MAT***a** *5FLAG-dia2-ΔLRR (his3MX) pep4Δ::ADE2*

Supplemental Table S1

Strains used in this study - all based on the W303 yeast genetic background, except for the yeast two-hybrid strain PJ69-4A.

Supplemental Experimental Procedures

Yeast Strains and Growth

The *Saccharomyces cerevisiae* strains that were used in this study are listed in Supplemental Table S1. Cells were grown in rich media (YPD) that contained yeast extract (1%), peptone (2%) and glucose (2%). As required, cells were synchronized in G1 by addition of 7.5 mg/ml alpha-factor mating pheromone and released into S phase by washing twice with fresh YPD media. To inhibit ribonucleotide reductase and slow progression through S-phase, hydroxyurea (HU; Sigma-Aldrich H8627) was added to a final concentration of 200mM. Cells were arrested in G2-phase by addition to the culture medium of 5μg/ml nocodazole (Sigma-Aldrich M1404). To induce degradation of Cdc48-aid, we added 0.5mM 3-indolacetic acid (IAA; I3750, Sigma-Aldrich) to the culture medium for the indicated time.

Yeast Two-Hybrid Assays

We performed two-Hybrid analysis by co-transformation of derivatives of pGADT7 (Gal4 activation domain; *LEU2* marker) and pGBKT7 (Gal4 DNA binding domain; *TRP1* marker) into the yeast strain PJ69-4A. Five independent transformed colonies were mixed together in PBS medium, before spotting ten-fold dilutions from 50,000 to 50 cells onto selective medium. We either used Synthetic Complete medium lacking tryptophan (to select for pGBKT7) and leucine (to select for pGADT7), or else used SC medium lacking tryptophan, leucine and histidine (additionally selective for the two-hybrid interaction).

Immunoprecipitation and immunoblotting of proteins from yeast cell extracts

After harvesting 250ml culture samples (about 2.5 x 10^9 cells), cell extracts were prepared at pH 7.9 or pH 9, in the presence of 100mM or 700mM potassium acetate, as indicated in the figures. Extracts were made as described previously [S1, S2], using a SPEX SamplePrep 6850 Freezer/Mill. In order to digest chromosomal DNA, extracts were incubated for 30 minutes at 4°C with 800 units of benzonase (71206-3, Merck Biosciences). Tagged proteins were isolated by immunoprecipitation with magnetic Dyna-beads M-270 Epoxy (Invitrogen) coupled to rabbit IgG (Sigma S-1265) or M2 anti-FLAG monoclonal antibody (Sigma F3165). Proteins were detected by immunoblotting using polyclonal antibodies previously described [S3], polyclonal anti-FLAG antibody (Sigma F-7425), polyclonal anti-Cdc53 antibody (Santa Cruz y-300, sc-50444), 9E10 anti-MYC antibody (Cancer Research UK) or with Peroxidase:Anti-Peroxidase complex (Sigma P-2026) for TAP-tagged proteins.

Purification of Ctf4 for *in vitro* complementation experiments

Ctf4-TAP was isolated from 2.5 g of frozen yeast as described above, using magnetic beads coupled to rabbit IgG. After a two-hour incubation with yeast extracts at 4˚C, the IgG-coated beads were washed twice with 1 ml of pH 9 wash buffer (100mM Tris-acetate pH 9, 100mM potassium acetate, 10mM magnesium acetate, 0.1% IGEPAL CA-360, 2mM sodium fluoride,

2mM sodium β-glycerophosphate pentahydrate) supplemented with protease inhibitors, once with 1 ml of pH 7.9 wash buffer (100mM HEPES-KOH pH 7.9, 100mM potassium acetate, 10mM magnesium acetate, 0.1% IGEPAL CA-360, 2mM sodium fluoride, 2mM sodium β-glycerophosphate pentahydrate), then once with 1ml of TEV cleavage buffer (100mM HEPES-KOH pH 7.9, 100mM potassium acetate, 10mM magnesium acetate, 0.1% IGEPAL CA-360). The magnetic beads were then agitated for one hour at 24°C in 80*μ*l of TEV cleavage buffer supplemented with 4*μ*l (40U) of AcTEV protease (12575015, Life Technologies). Following elution, the supernatant was removed and 40*μ*l of supernatant was added to each millilitre of the recipient cell extract as indicated (the negative control comprised 40*μ*l of TEV cleavage buffer).

Supplemental References

S1. De Piccoli, G., Katou, Y., Itoh, T., Nakato, R., Shirahige, K., and Labib, K. (2012). Replisome stability at defective DNA replication forks is independent of S phase checkpoint kinases. Molecular cell *45*, 696-704.

S2. Maric, M., Maculins, T., De Piccoli, G., and Labib, K. (2014). Cdc48 and a ubiquitin ligase drive disassembly of the CMG helicase at the end of DNA replication. Science *346*, 1253596.

S3. Gambus, A., Jones, R.C., Sanchez-Diaz, A., Kanemaki, M., van Deursen, F., Edmondson, R.D., and Labib, K. (2006). GINS maintains

association of Cdc45 with MCM in replisome progression complexes at eukaryotic DNA replication forks. Nat Cell Biol *8*, 358-366.
